# ERCC6L facilitates the progression of laryngeal squamous cell carcinoma by the binding of FOXM1 and KIF4A

**DOI:** 10.1038/s41420-023-01314-3

**Published:** 2023-02-02

**Authors:** Meng Cui, Yu Chang, Jiheng Wang, Junfu Wu, Gang Li, Jie Tan

**Affiliations:** 1 Department of Head and Neck Thyroid, The Affiliated Cancer Hospital of Zhengzhou University & Henan Cancer Hospital, 127 Dongming Road, Zhengzhou, 450008 People’s Republic of China; 2 Department of Oncology, The First Affiliated Hospital of Zhengzhou University, 1 Jianshe Dong Road, Zhengzhou, 450007 People’s Republic of China; 3 Department of Otorhinolaryngology Head and Neck Surgery, Peking University People’s Hospital, Peking University, Xi Zhi Men South Street 11, Western District, Beijing, 100034 P.R. China

**Keywords:** Head and neck cancer, Cell biology, Molecular biology

## Abstract

The role of excision repair cross-complementation group 6-like (ERCC6L) has been reported in several cancers, but little is known about its expression and function in laryngeal squamous cell carcinoma (LSCC). In this study, the expression of ERCC6L in LSCC was determined by immunohistochemistry and its correlation with prognostic factors was analyzed. Furthermore, cytological functional validation elucidated the role and underlying mechanisms of ERCC6L dysregulation in LSCC. Our data revealed that ERCC6L expression was elevated in LSCC and it’s correlated with TNM stage. In addition, ERCC6L knockdown LSCC cells showed decreased proliferation and migration, increased apoptosis, and reactive oxygen species (ROS). Mechanically, overexpression of ERCC6L promoted nuclear translocation of FOXM1 to facilitate direct binding to the KIF4A promoter and upregulated KIF4A expression. Furthermore, KIF4A knockdown attenuated the role of ERCC6L overexpression in promoting proliferation, migration, and tumorigenesis of LSCC cells. In summary, ERCC6L promoted the binding of FOXM1 and KIF4A in LSCC cells to drive their progression, which may be a promising target for precision therapy in this disease.

## Introduction

Laryngeal squamous cell carcinoma (LSCC) is one of the most common subtypes of laryngeal cancer. It is aggressive and has relatively high morbidity and mortality [1]. In addition, the onset of LSCC is insidious, with approximately 60% of patients diagnosed in advanced (III or IV) stage [2]. Despite substantial improvements in clinical management methods such as radiotherapy, chemotherapy, and surgery, the 5-year overall survival in LSCC has not improved significantly over the past 20 years [3]. In recent years, new targeted drugs have the hope of improving the prognosis of LSCC patients in the metastatic environment [4]. Therefore, the unraveling of the pathogenesis of LSCC is urgently needed to identify its diagnostic biomarkers and effective new therapeutic targets.

Excision repair cross-complementation Group 6-Like (ERCC6L) belongs to the SNF2 helicase-like ATPase family, also known as PICH (Plk1 Interacting Checkpoint Helicase) [5]. Deletion of ERCC6L in human or animal cells can lead to marked chromosomal abnormalities, DNA damage, embryonic lethality, apoptosis and TP53 activation [6]. Therefore, ERCC6L is closely related to cell mitosis and chromatin remodeling. In view of this, the correlation between ERCC6L and tumorigenesis has been concerned and explored. For example, ERCC6L overexpression is associated with disease progression and poor survival in patients with breast, renal, and hepatocellular carcinoma [7, 8]. In addition, ERCC6L silencing can lead to cycle arrest, proliferation inhibition, and reduced invasion of tumor cells in renal carcinoma, breast cancer, colorectal cancer, hepatocellular carcinoma, and non-small cell lung adenocarcinoma [9–13]. Thus, the above evidence demonstrated that the expression of ERCC6L was dysregulated in many cancers, which may play a crucial role. However, the expression and role of ERCC6L in LSCC remains to be investigated.

In this study, the expression of ERCC6L in LSCC was determined by immunohistochemistry, and its correlation with prognostic factors was analyzed. Furthermore, cytological functional validation elucidated the role and underlying mechanisms of ERCC6L dysregulation in LSCC.

## Results

### ERCC6L is abundantly highly expressed in LSCC

**Fig. 1:**
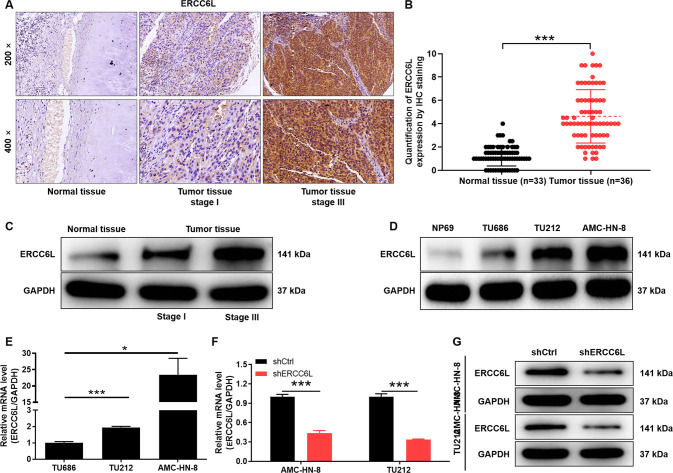
Expression detection of ERCC6L in LSCC. **A** Representative IHC staining of ERCC6L expression in LSCC tissues and normal tissues. **B** Quantitative IHC results of ERCC6L expression in LSCC tissue (*n* = 36) and adjacent normal tissue (*n* = 33). **C** The mRNA expression of ERCC6L in LSCC cells such as TU686, TU212, and AMC‐HN‐8 was detected using qPCR. **D** The mRNA expression of ERCC6L was detected after shRNA targeting ERCC6L (shERCC6L) was transduced into AMC-HN-8 and TU212 cells. **E** The protein levels of ERCC6L in shERCC6L-mediated AMC-HN-8 and TU212 cells was analyzed by western blotting. **F** The mRNA expression of ERCC6L was detected after shRNA targeting ERCC6L (shERCC6L) was transduced into AMC-HN-8 and TU212 cells. **G** The protein expression of ERCC6L was detected after shERCC6L was transduced into AMC-HN-8 and TU212 cells. The representative images were selected from at least three independent experiments. Data was shown as mean ± SD. *P < 0.05, ***P < 0.001.

Tissue microarrays composed of LSCC tissue (*n* = 36) and adjacent normal tissue (*n* = 33) were used for IHC staining to reveal differences in their ERCC6L expression. The typical IHC images showed large areas of dark brown in tumor tissue, but little in normal tissue (Fig. 1A). Quantitative results based on IHC staining indicated that the scores in tumor tissues were significantly higher than those in normal tissues (*P* < 0.001, Fig. 1B). Scores higher than the median (4.5) were considered to ERCC6L high expression, otherwise low ERCC6L expression. Statistics showed that ERCC6L was highly expressed in 52.8% of tumor tissues, while all normal tissues were low ERCC6L expression (*P* < 0.001, Table 1). Consistently, the results of WB confirmed the high expression of ERCC6L in LSCC (Fig. 1C). Moreover, ERCC6L was abundantly highly expressed in LSCC cells, such as TU686, TU212, and AMC‐HN‐8 (Fig. 1D, E). The above results revealed that ERCC6L was generally highly expressed in LSCC. Next, the correlation between ERCC6L expression and clinicopathological features of LSCC patients was preliminarily analyzed using Mann-Whitney U. Of note, pathological staging criteria for LSCC patients were based on the seventh edition of the American Joint Committee on Cancer (AJCC) Cancer Staging Manual. Our data indicated that the expression level of ERCC6L was significantly positively correlated with T infiltrate and TNM of LSCC (*P* < 0.001, Table 2). Consistently, the analysis results of Spearman correlation coefficient further confirmed the above data (*P* < 0.001, Table 3). Taken together, the expression of ERCC6L was elevated in LSCC and correlated with poor prognostic factors, suggesting that ERCC6L may be a diagnostic marker for this disease.

**Table 1:** Expression patterns in laryngocarcinoma tissues and normal tissues was revealed by immunohistochemistry analysis.

ERCC6L expression	Tumor tissue		Normal tissue		*p* value
	Cases	Percentage	Cases	Percentage	
Low	17	47.2%	33	100.0%	<0.001
High	19	52.8%	0	0%

**Table 2:** Relationship between ERCC6L expression and tumor characteristics in patients with laryngocarcinoma.

Features	No. of patients	ERCC6L expression		*p* value
		Low	High	
All patients	36	17	19	
Age (years)				1.000
<62	17	8	9	
≥62	19	9	10	
Gender				0.778
Male	35	16	19	
Female	1	1	0	
T Infiltrate				0.001
T1	8	8	0	
T2	16	8	8	
T3	7	1	6	
T4	5	0	5	
Lymphatic metastasis (*N*)				0.175
N0	23	13	10	
N1	4	2	2	
N2	9	2	7	
Maximum tumor diameter				0.271
≤2 cm	17	10	7	
>2 cm	19	7	12	
TNM				0.001
I	7	7	0	
II	7	5	2	
III	9	3	6	
IV	13	2	11

**Table 3:** Relationship between ERCC6L expression and tumor characteristics in patients with laryngocarcinoma.

		ERCC6L
T Infiltrate	Spearman correlation coefficient	0.672
	Significance (two tails)	0.000
	*N*	36
TNM	Spearman correlation coefficient	0.638
	Significance (two tails)	0.000
	*N*	36

### ERCC6L drives progression of LSCC cells

**Fig. 2:**
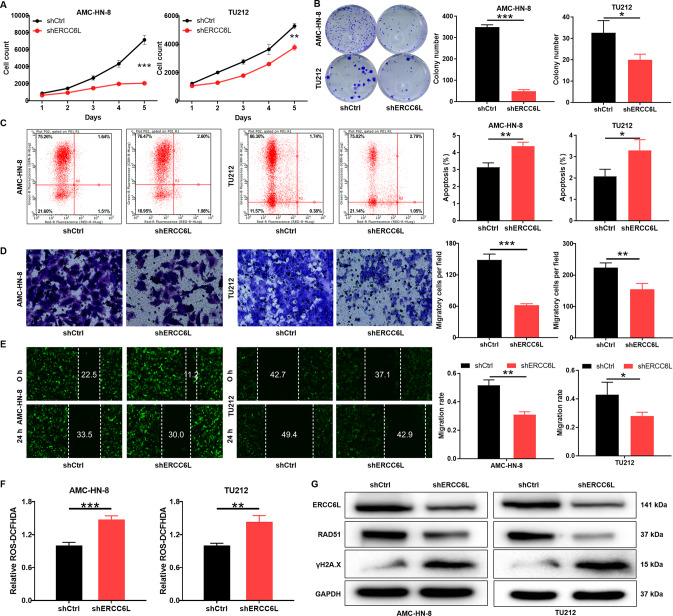
ERCC6L knockdown inhibits LSCC progression in vitro. **A** Celigo cell counting assay was employed to show the effects of ERCC6L on cell proliferation of AMC-HN-8 and TU212 cells. **B** Colony forming ability of the shERCC6L-mediated AMC-HN-8 and TU212 cells was detected. **C** Flow cytometry was performed to detect cell apoptosis of AMC-HN-8 and TU212 cells with or without ERCC6L knockdown. The shERCC6L-mediated LSCC cell migration ability was accessed by (**D**) Transwell assay and (**E**) wound-healing assay. **F** The ROS content in the shCtrl group and shERCC6L group was analyzed by Reactive Oxygen Species (ROS) assay. **G** The protein levels of RAD51 and γH2A.X in AMC-HN-8 and TU212 cells was analyzed by western blotting. The representative images were selected from at least 3 independent experiments. Data was shown as mean ± SD. *P < 0.05, **P < 0.01, ***P < 0.001.

ERCC6L expression was detected after shRNA targeting ERCC6L (shERCC6L) was transduced into AMC-HN-8 and TU212 cells. The mRNA level of ERCC6L in the shERCC6L group was significantly lower than that in the shCtrl group in LSCC cells (*P* < 0.001, Fig. 1F). As expected, protein level of ERCC6L in shERCC6L-mediated AMC-HN-8 and TU212 cells was decreased relative to shCtrl (Fig. 1G). Subsequently, the effects of ERCC6L on LSCC cell proliferation, clone formation, apoptosis, and migration were assessed by loss-of-function assays in vitro. As shown in Fig. 2A, the cell proliferation ability of AMC-HN-8 and TU212 in the shERCC6 group was attenuated compared to shCtrl (*P* < 0.01). Not surprisingly, LSCC cells in the shERCC6 group formed fewer and smaller cell clones compared to shCtrl (*P* < 0.05, Fig. 2B). Furthermore, flow cytometry-based data indicated that AMC-HN-8 and TU212 cells showed a stronger apoptosis rate after ERCC6 was stably knocked down (*P* < 0.05, Fig. 2C). In addition, the migration ability of LSCC cells in shCtrl and shERCC6 groups was detected by Transwell and wound healing assays, respectively. The number of crystal violet-stained cells in the shERCC6 group was significantly less than that of shCtrl, suggesting that knockdown of ERCC6 inhibited the migration of LSCC cells (*P* < 0.01, Fig. 2D). Undoubtedly, wound healing experiments showed the same phenomenon, confirming that ERCC6 may drive the migration of LSCC cells to some extent (*P* < 0.05, Fig. 2E). Interestingly, ROS have been detected in nearly all cancers, and they contribute to tumor development and progression [14]. The present study indicated that the ROS content in the shERCC6L group was increased compared to the shCtrl group (*P* < 0.01, Fig. 2F). Moreover, RAD51 and γH2A.X are involved in ROS generation and redox stress [15, 16]. Our results indicated that knockdown of ERCC6L downregulated RAD51 and upregulated γH2A.X (Fig. 2G). Collectively, ERCC6L drove progression of LSCC cells.

### ERCC6L promotes the binding of FOXM1 and KIF4A in LSCC cells

**Fig. 3:**
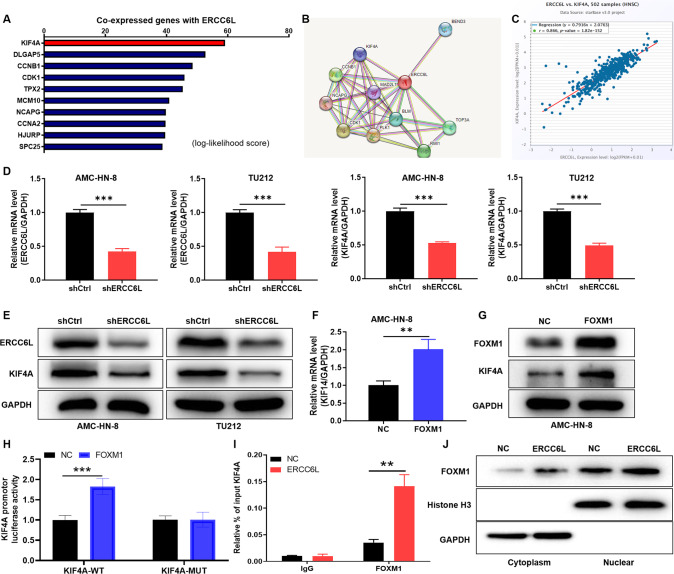
ERCC6L promotes the binding of FOXM1 and KIF4A in LSCC cells. **A** Bioinformatics analysis revealed that KIF4A and ERCC6L were co-expressed genes. **B** We used the string online database (https://cn.string-db.org/) to analyze and predict the proteins interacting with ERCC6L, and found that KIF4A interacted with ERCC. **C** The expression levels of KIF4A and ERCC6L were significantly positively correlated. **D**, **E** The expression of ERCC6L and KIF4A at mRNA and protein levels in shERCC6L-mediated AMC-HN-8 and TU212 cells was analyzed. **F**, **G** The expression of KIF4A at mRNA and protein levels in FOXM1-mediated AMC-HN-8 cells was analyzed. **H** The wild-type (WT) and mutant (MUT) KIF4A promoter regions were constructed in HEK293T cells and the dual-luciferase activity of KIF4A was analyzed. **I** We performed Ch-IP assay and verified that ERCC6L overexpression can promote the binding of transcription factor FOXM1 to the promoter region of KIF4A. **J** The protein expression of FOXM1 was detected in the cytoplasm and nucleus of ERCC6L-overexpressing AMC-HN-8 cells. WB analysis showed that ERCC6L overexpression promoted nuclear translocation of FOXM1. The representative images were selected from at least three independent experiments. Data was shown as mean ± SD. **P < 0.01, ***P < 0.001.

Bioinformatics analysis revealed that KIF4A and ERCC6L were co-expressed genes (Fig. 3A). Moreover, we used the string online database (https://cn.string-db.org/) to analyze and predict the proteins interacting with ERCC6L, and found that KIF4A interacted with ERCC6 (Fig. 3B). In addition, the expression levels of KIF4A and ERCC6L were significantly positively correlated (Fig. 3C). To further verify the effect of ERCC6L on KIF4A, knock downed ERCC6L in LSCC cells to detect the expression of KIF4A at mRNA and protein levels. The results indicated that knockdown of ERCC6L downregulated the expression of KIF4A at mRNA and protein levels (*P* < 0.001, Fig. 3D, E). On the other hand, KIF4A had been demonstrated to be a direct transcriptional target of FOXM1, which binds to the KIF4A promoter [17]. In view of the above results, we conducted further verification. Our data showed that FOXM1 overexpression upregulated the expression of KIF4A (*P* < 0.001, Fig. 3F, G). Furthermore, the wild-type (WT) and mutant (MUT) KIF4A promoter regions were constructed in HEK293T cells according to the predicted binding sites (2Kb upstream of the TSS site, chrX: 70288104-70420886) of FOXM1 and KIF4A. The results showed that FOXM1 overexpression enhanced the dual-luciferase activity of WT-KIF4A but not MUT-KIF4A, suggesting that FOXM1 directly associated with the KIF4A promoter (*P* < 0.001, Fig. 3H). In addition, we performed Ch-IP assay and verified that ERCC6L overexpression can promote the binding of transcription factor FOXM1 to the promoter region of KIF4A (*P* < 0.01, Fig. 3I). More interestingly, WB analysis showed that ERCC6L overexpression promoted nuclear translocation of FOXM1 (Fig. 3J). Collectively, our findings supported the view that overexpression of ERCC6L promoted nuclear translocation of FOXM1 to facilitate direct binding to the KIF4A promoter and upregulated KIF4A expression.

### ERCC6L drives LSCC progression dependent on the presence of KIF4A

**Fig. 4:**
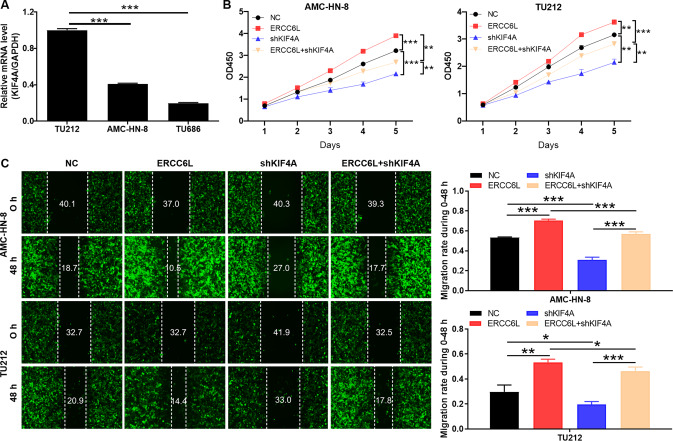
ERCC6L drives LSCC progression dependent on the presence of KIF4A. **A** The mRNA expression of KIF4A in LSCC cells such as TU686, TU212, and AMC‐HN‐8 was detected using qPCR. **B**, **C** ERCC6L overexpression and KIF4A knockdown were separately constructed in AMC-HN-8 and TU212 cells for loss/gain function assays. The cells models were subjected to the (**B**) MTT assay and (**C**) wound-healing assay. The representative images were selected from at least three independent experiments. Data was shown as mean ± SD. *P < 0.05, **P < 0.01, ***P < 0.001.

Given that the molecular mechanisms of ERCC6L and KIF4A had been initially revealed, their coordinated roles in cells required further verification. Firstly, the data indicated that KIF4A was abundantly expressed in LSCC cells (Fig. 4A). Subsequently, ERCC6L overexpression and KIF4A knockdown were separately constructed in AMC-HN-8 and TU212 cells for loss/gain function assays. As shown in Fig. 4B, the cell proliferation ability of AMC-HN-8 and TU212 in the shKIF4A group was attenuated (*P* < 0.01), but enhanced in the ERCC6L overexpression group compared to NC (*P* < 0.01). Moreover, KIF4A knockdown can alleviate the promoting effect of ERCC6L overexpression on LSCC cell proliferation (*P* < 0.01, Fig. 4B). Not surprisingly, ERCC6L overexpression enhanced the migration of LSCC cells, and this effect was partially reversed by KIF4A knockdown (*P* < 0.05, Fig. 4C). Taken together, ERCC6L promoted LSCC progression may dependent on the presence of KIF4A.

### KIF4A knockdown attenuates the role of ERCC6L overexpression in promoting tumorigenesis of LSCC cells

**Fig. 5:**
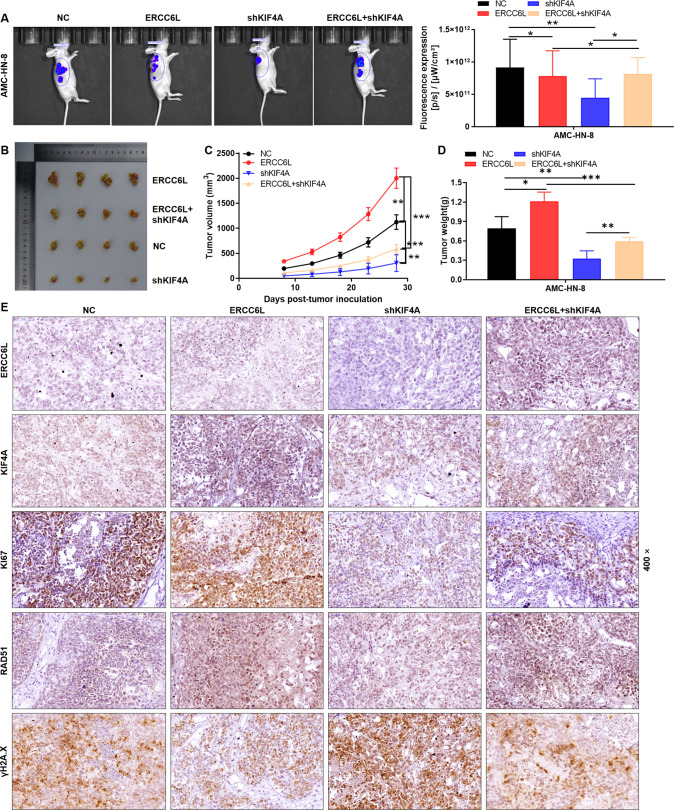
KIF4A knockdown attenuates the role of ERCC6L overexpression in promoting tumorigenesis of LSCC cells. **A** In vivo imaging was performed to evaluate the tumor burden in mice of NC, ERCC6L, shKIF4A, and ERCC6L + shKIF4A groups post tumor-inoculation. **B**–**D** Mice were monitored for 28 days and data were collected on the volume and weight of the xenografts. **E** IHC staining was performed to determine the expression of ERCC6L, KIF4A, Ki67, RAD51, and γH2A.X. Data was shown as mean ± SD (*n* = 4). *P < 0.05, **P < 0.01, ***P < 0.001.

In vivo mice xenografts were established to further validate the role of ERCC6L and KIF4A in regulating LSCC. The mice were subcutaneously inoculated with AMC-HN-8 cells and divided into the following four groups: NC, ERCC6L, shKIF4A and ERCC6L + shKIF4A. Mice were monitored for 28 days and data were collected on the intensity of fluorescence expression of the xenografts. Figure 5A showed the fluorescent pictures of the tumors of mice in each group on day 28. The fluorescence intensity of tumor was ERCC6L group, ERCC6L + shKIF4A group and shKIF4A group in order from high to bottom. Consistently, the same was true for the size of tumors taken from mice (Fig. 5B). On day 28, the mean tumor volume was 1125.08 mm^3^ in the ERCC6L group, only 305.02 mm^3^ in the shKIF4A group, and 589.77 mm^3^ in the ERCC6L + shKIF4A group (Fig. 5C). As expected, tumor weight showed the same trend (Fig. 5D). In addition, IHC staining was performed to determine the expression of ERCC6L, KIF4A, Ki67, RAD51 and γH2A.X (Fig. 5E). These results are consistent with in vitro data, suggesting that KIF4A knockdown attenuated the role of ERCC6L overexpression in promoting tumorigenesis of LSCC cells.

## Discussion

LSCC has relatively high morbidity and mortality, and current clinical treatment options are limited [3]. Therefore, the elucidation of the molecular mechanism of LSCC progression and the identification of potential therapeutic targets provide theoretical support for clinical treatment. On the other hand, the role of ERCC6L has been reported in several cancers, but little is known about its expression and function in LSCC. In this study, we found that ERCC6L was significantly upregulated in LSCC and was positively associated with clinicopathological features, such as T infiltrate and TNM. Taken together, our findings suggested that ERCC6L may be a diagnostic marker for this disease.

However, the exact mechanism by which ERCC6L regulated tumor cell development and progression in LSCC remained unclear. The present study demonstrated that ERCC6L knockdown LSCC cells showed decreased proliferation and migration, and increased apoptosis. Interestingly, ROS have been detected in nearly all cancers, and they contribute to tumor development and progression [14]. Moreover, RAD51 and γH2A.X are involved in ROS generation and redox stress [15, 16]. Our results indicated that knockdown of ERCC6L increased ROS content, upregulated γH2A.X and downregulated RAD51 in LSCC cells. These results suggested that ERCC6L was involved in ROS generation in LSCC cells and contributed to the malignant progression of this tumor cells.

Bioinformatics analysis revealed that KIF4A and ERCC6L were co-expressed genes. In addition, the expression levels of KIF4A and ERCC6L were significantly positively correlated. KIF4A had been demonstrated to be a direct transcriptional target of Forkhead Box M1 (FOXM1), which binds to the KIF4A promoter [17]. FOXM1 is an important regulator of many biological processes, and dysregulation of FOXM1 leads to carcinogenesis and tumor progression [18]. In view of the above results, we conducted further verification. Our data showed that FOXM1 overexpression upregulated the expression of KIF4A. In addition, we performed dual-luciferase assay and Ch-IP assay, verifying that ERCC6L overexpression can promote the binding of transcription factor FOXM1 to the promoter region of KIF4A. More interestingly, it has been reported that FOXM1d is located in the cytoplasm and does not directly control transcription [19]. In this study, our data showed that ERCC6L overexpression promoted nuclear translocation of FOXM1. Collectively, our findings supported the view that overexpression of ERCC6L promoted nuclear translocation of FOXM1 to facilitate direct binding to the KIF4A promoter and upregulated KIF4A expression.

KIF4A is overexpressed in most tumors, but also low in a minority [20]. Yang et al., found that KIF4A is abnormally highly expressed in human clear cell renal cell carcinoma tissues and can act as a tumor-inducing gene [21]. Zhu et al., proposed that enhanced KIF4A expression in osteosarcoma predicts poor prognosis and promotes tumor growth by activating the MAPK pathway [22]. Cao et al., demonstrated that KIF4A plays an important role in the progression of CRPC and is a key determinant of CRPC resistance to endocrine therapy [23]. In this study, the results indicated that KIF4A knockdown attenuated the role of ERCC6L overexpression in promoting proliferation, migration, and tumorigenesis of LSCC cells. Therefore, our data together with previous studies further confirmed the critical role of FOXM1c in cancer cell progression. In summary, ERCC6L promoted the binding of FOXM1 and KIF4A in LSCC cells to drive their progression, which may be a promising target for precision therapy in this disease.

## Materials and methods

### Tissue microarray and immunohistochemistry (IHC) staining

This study was approved by the Research Ethics Committee of Affiliated Cancer Hospital of Zhengzhou University & Henan Cancer Hospital, and written informed consent was obtained from each participant. LSCC tissue (*n* = 36) and adjacent normal tissue (*n* = 33) constitute a tissue microarray for IHC staining. In detail, the tissue microarray was soaked in xylene and alcohol in turn for dewaxing and rehydration. After that, the tissue microarray was boiled in sodium citrate buffer (pH = 6.0) for antigen repair. At room temperature, they were incubated with 5% animal serum in PBST for 30 min, then incubated with the primary antibody (anti-ERCC6L, 1:200, Abcam, USA) for another 2 h. Tissue microarray and secondary antibody (goat anti-rabbit IgG, 1:400, Beyotime, USA) were incubated at 4 °C for 2 h, washed with PBST, and then stained with DAB and hematoxylin, respectively. Finally, the tissues were observed with microscopic and IHC scores were determined by staining percentage scores (classified as: 1 (1–24%), 2 (25–49%), 3 (50–74%), 4 (75–100%)) and staining intensity scores (scored as 0: signal less color, 1: brown, 2: light yellow, 3: dark brown).

### Cell culture

Human laryngeal cancer cells (TU686, TU212 and AMC‐HN‐8) were obtained from Cell Bank of the Chinese Academy of Sciences (Shanghai, China) and were kept in a humidified incubator at 37 °C under an atmosphere with 5% CO_2_ in air. The cells were cultured in Dulbecco’s modified Eagle’s medium (DMEM, HyClone) containing 10% fetal bovine serum (FBS, Invitrogen). Of note, all cells were validated by STR.

### Lentiviral transduction

The small hairpin RNA sequences targeting ERCC6L (shERCC6: 5′-CTGCCCAAAGAGGGTGAGAAA-3′, 5’-TAAAGAAGACGTACAGAAGAA-3′, 5′-CAACTAAAGGATGATGAGATT-3′), KIF4A (shKIF4A 5′-TATACTGCAGAGCAAGAGAAT-3′, 5′-ATTGATACTGCGGTGGAGCAA-3′, 5′-CTTACTGAAGTGCGTGGTCAA-3′) and Scramble sequence (negative control, shCtrl: 5′-TTCTCCGAACGTGTCACGT-3′) were ligated into BR-V-108 lentiviral vector (Shanghai Yiberui Biomedical Technology), respectively. The packaged lentivirus was transduced into AMC-HN-8 and TU212 cells using Lipofectamine 2000 (Invitrogen) in accordance with the manufacturer’s protocol. After cultured for 72 h at 37 °C, green fluorescent protein (GFP) expression was detected under a microscope and stably transduced cell lines were selected with puromycin [24].

### Quantitative real‐time PCR (qRT‐PCR)

The cells total RNA was extracted using Trizol according to the operating instructions (Sigma, Cat. No. T9424-100m). Hiscript QRT supermix (Vazyme, Cat. No. R123-01) was used for reverse transcription of RNA to obtain cDNA. SYBR Green mastermixs (Vazyme, Cat. No. Q111-02), target primers, and cDNA were used to configure the reaction system of qPCR. The relative mRNA expression level of the target in each sample relative to the control group was reflected by 2^−△Ct^. The upstream and downstream primer sequences for amplifying ERCC6L are shown below: 5′-AAAAGTCAAGCAACCCAGAGG-3′ and 5′-GTAAAGGCACAAGTCGTATCCA-3′; The upstream and downstream primer sequences for amplifying KIF4A are shown below: 5′- CTGCCAACAAGCGTCTCAAGG-3′ and 5′-CCTTCCATTCCACGGCTCTGA-3′; The upstream and downstream primer sequences of the internal reference GAPDH are shown below: 5’-TGACTTCAACAGCGACACCCA-3′ and 5′-CACCCTGTTGCTGTAGCCAAA-3′.

### Western blotting (WB) analysis

AMC-HN-8 and TU212 cells protein was extracted with 1× cell lysis buffer (Promega, USA) and protein concentrations were quantified by BCA Protein Assay Kit (Pierce, USA). The 20 µg proteins in each group were separated by 10% SDS-PAGE (Invitrogen) and then transferred onto PVDF membranes. After the PVDF membrane was sealed by the blocking solution (TBST solution containing 5% skim milk) at room temperature for 1 h, incubated with the primary antibody (anti-ERCC6L, 1:1000, Abcam, USA; anti-KIF4A, 1:1000, Abcam, USA; anti-RAD51, 1:1000, Proteintech, China; anti-γH2A.X, 1:1000, Abcam, USA; GAPDH, 1:30000, Proteintech, China) overnight at 4 °C. After the membrane was washed by TBST, the secondary antibody (goat anti-rabbit IgG, 1:3000, Beyotime, USA) was added and incubated at room temperature for 2 h. Finally, the blots were visualized using enhanced chemiluminescence (ECL) (Amersham).

### Celigo cell counting assay

AMC-HN-8 and TU212 cells were laid with 96-well plates at a density of 2000 cell/well. From the second day after laying, the Celigo reading board was tested once a day for 5 days continuously. The data were statistically plotted, and the cell proliferation curve for 5 days was drawn.

### MTT assay

AMC-HN-8 and TU212 cells were laid with 96-well plates at a density of 2000 cell/well. From the second day after laying, 20 μL 5 mg/mL MTT (Genview, China, Cat. No. JT343) was added into the well 4 h before the end of culture. After 4 h, the culture medium was completely absorbed, and 100 μL DMSO solution was added. The OD value of 450/570 nm was detected by Microplate Reader (Tecan infinite).

### Colony formation assay

AMC-HN-8 and TU212 cells were cultured in 6-well plates at a density of 800 cell/well for 14 days. Notably, a single cell lasts for more than 6 generations in vitro, and the cell population composed of its progeny is called a clone. At this point each clone contained more than 50 cells, ranging in size from 0.3 to 1.0 mm. Subsequently, 4% paraformaldehyde of 1 mL was added to fix cells for 30 min. Later, 500 μL GIEMSA staining solution was used to dye the cells for 20 min and photographed the cell clones.

### Cell apoptosis analysis by flow cytometry

AMC-HN-8 and TU212 cells were inoculated in 6-well plate (2 ml/well) and cultured continuously for 5 days. The cells were then centrifuged for 5 min, cell precipitates were washed by D-Hanks (pH = 7.2–7.4) precooled at 4 °C. After the cell precipitation was resuscitated by 200 μL 1 × binding buffer, 10 μL Annexin V-APC was added for cell staining. Cell apoptosis rate was calculated by flow cytometry in 3 randomly selected visual fields.

### Transwell assay

AMC-HN-8 and TU212 cells were inoculated on well-hydrated chamber (3422 corning) at a density of 50,000 cell/well. The inner chamber contains 100 μL serum-free medium and the outer chamber contains 600 μL containing 30% FBS. 100 μL of cell suspension was diluted in serum-free medium and then added to each compartment for 24 h. The migrated cells were fixed by 4% formaldehyde and photographed after Giemsa staining to analyze the cell migration ability.

### Wound-healing assay

AMC-HN-8 and TU212 cells were inoculated on 96-well plate (100 μL/well) at a density of 50,000 cell/well. The next day, the low concentration serum medium was replaced, and a scratch meter was used to aim at the center of the lower end of the 96-well plate and nudge upward to form scratches. Cellomics (Thermo) was used to scan the plate and analyze the migration area when they were continuously cultured for 0, 24, and 48 h, respectively. The migration rate was calculated based on the cell migration distance using NIH image software.

### Reactive oxygen species (ROS) assay

ROS was detected using the fluorescent probe DCFH-DA following the steps provided in the kit (Beyotime Biotechnology). AMC-HN-8 and TU212 cells were suspended in DCFH-DA diluted with serum-free medium (1:1000) and incubated in a cell incubator at 37 °C for 20 min. Probes and cells were mixed by shaking them every 3–5 min. Subsequently, cells were washed three times with serum-free cell culture medium to sufficiently remove excess DCFH-DA and detected by fluorescence microplate reader at OD488.

### Dual-luciferase assay

Promoter deletion was analyzed using a dual-luciferase reporting system as previously described [25]. The KIF14A promoter region fragment (2Kb upstream of the TSS site, chrX: 70288104-70420886) was amplified and cloned into the luciferase reporter vector GL002 (Promega Madison, USA), designated as GL002-KIF14A. Mutant construct KIF14A-MUT was generated by site-directed mutagenesis and KIF14A-WT as negative control. According to the instructions of Promega dual-luciferase system (Cat. No. E2940, Madison, USA), Firefly luciferase value and Renilla luciferase signals were determined.

### Chromatin immunoprecipitation (ChIP)-qPCR assay

The ChIP-qPCR assay was performed as described previously [26]. AMC-HN-8 cells with ERCC6L overexpression were cross-linked with formaldehyde, lysed in the SDS buffer and sheared mechanically by sonication to fragment the DNA. Protein–DNA complexes were precipitated with control goat anti-rabbit IgG (Sigma, USA), Histone H3 (D2B12) XP^®^ Rabbit mAb (CST), and anti-FOXM1 (1:100, Proteintech), respectively. After separating the complex from the antibody, using the primers specific for KIF14A promoter and SYBR premix (Vazyme) to detect the eluted DNA fragment. The primer sequence for KIF14A as follows: 5′- CTGCCAACAAGCGTCTCAAGG-3′ and 5′-CCTTCCATTCCACGGCTCTGA-3′.

### Animal xenograft model

The animal experiment was approved and performed according to the guidelines of Affiliated Cancer Hospital of Zhengzhou University & Henan Cancer Hospital. Male BALB/c-nu mice (4-weeks old, *n* = 16) were purchased from Shanghai Lingchang Biotechnology Co., Ltd. (Shanghai, China). After 1 week of adaptive feeding, mice were subcutaneously inoculated with 1 × 10^7^ AMC-HN-8 cells to establish a xenograft model and divided into the following four groups: NC (*n* = 4), ERCC6L (*n* = 4), shKIF4A (*n* = 4) and ERCC6L + shKIF4A (*n* = 4). After a week, data on mouse body weight and tumor size (tumor volume: π/6 × length × width× width) were collected every 5 days. After continuous feeding for 28 days, the mice were anesthetized by intraperitoneal injection of 0.7% pentobarbital sodium (10 μL/g) and under the IVIS Spectrum (Perkin Elmer) for fluorescence imaging observation. Subsequently, the mice were sacrificed by cervical vertebrae and the tumors were removed for tissue sections. Tissue sections were stained by IHC to reveal protein expression as previously described. Antibodies were used as follows: primary antibody (anti-ERCC6L, 1:200, Abcam, USA; anti-KIF4A, 1:200, Abcam, USA; anti-KI67, 1:200, Abcam, USA; anti-RAD51, 1:200, Proteintech Group, USA; anti-YH2AX, 1:200, Abcam, USA) and secondary antibody IgG (1:400, Abcam, USA).

### Statistical analysis

The data came from three separate experiments, expressed as mean ± SD. The significance differences between groups were determined using the two-tailed Student’s t test or One-way ANOVA analysis. Statistical analyses and graphs were performed by GraphPad Software 8.0 and *P* value < 0.05 as statistically significant.

### Supplementary information

Original Data File

Original Data File

Original Data File

## Data Availability

Data will be made available on request.
