# Correction: ERCC6L facilitates the progression of laryngeal squamous cell carcinoma by the binding of FOXM1 and KIF4A

**DOI:** 10.1038/s41420-023-01609-5

**Published:** 2023-08-29

**Authors:** Meng Cui, Yu Chang, Jiheng Wang, Junfu Wu, Gang Li, Jie Tan

**Affiliations:** 1 Department of Head and Neck Thyroid, The Affiliated Cancer Hospital of Zhengzhou University & Henan Cancer Hospital, 127 Dongming Road, Zhengzhou, 450008 People’s Republic of China; 2 Department of Oncology, The First Affiliated Hospital of Zhengzhou University, 1 Jianshe Dong Road, Zhengzhou, 450007 People’s Republic of China; 3 Department of Otorhinolaryngology Head and Neck Surgery, Peking University People’s Hospital, Peking University, Xi Zhi Men South Street 11, Western District, Beijing, 100034 P.R. China

**Keywords:** Head and neck cancer, Cell biology, Molecular biology

Correction to: *Cell Death Discovery* 10.1038/s41420-023-01314-3, published online 02 February 2023

In this article the affiliation of Jie Tan (Affiliation 3) is given erroneously as “Department of Otorhinolaryngology Head and Neck Surgery, Peking University People’s Hospital, Peking University, 133 Fuchengmennei Dajie, Beijing, 100044, P.R. China.”

it should read:

“Department of Otorhinolaryngology Head and Neck Surgery, Peking University People’s Hospital, Peking University, Xi Zhi Men South Street 11, Western District, Beijing, 100034, P.R. China.”

The original article has been corrected.

