# Supplementary material for: ERCC6L facilitates the progression of laryngeal squamous cell carcinoma by the binding of FOXM1 and KIF4A
Source: Cell Death Discov. 2023 Feb 2;9:41. doi: 10.1038/s41420-023-01314-3 (PMC9892579; doi:10.1038/s41420-023-01314-3)
Supplement: Supplementary file 1 — Original Data File [file 41420_2023_1314_MOESM1_ESM.pdf]

Fig 1E-AMCHN-8
ERCC6L

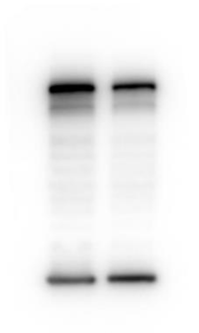

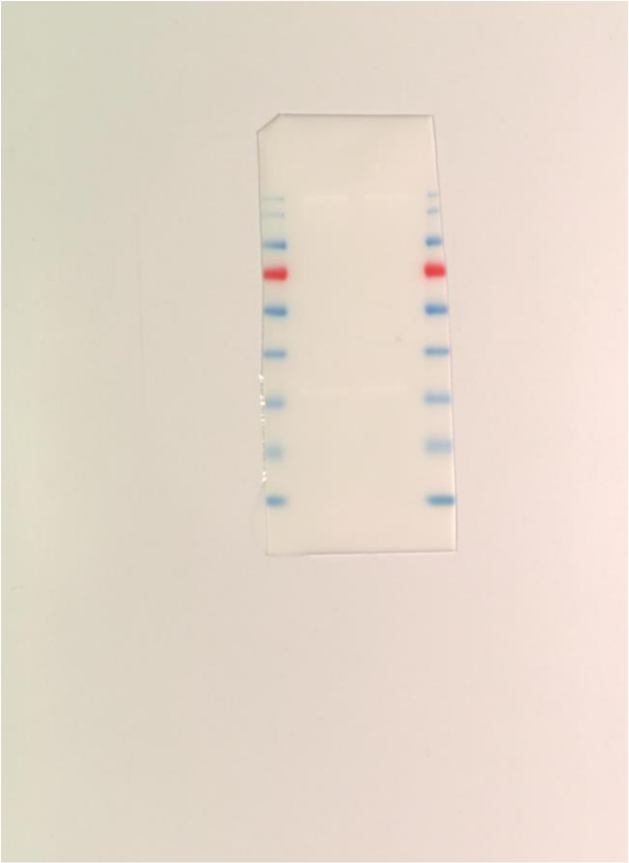

Fig 1E-AMCHN-8 GAPDH

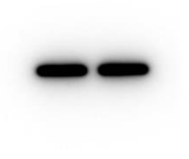

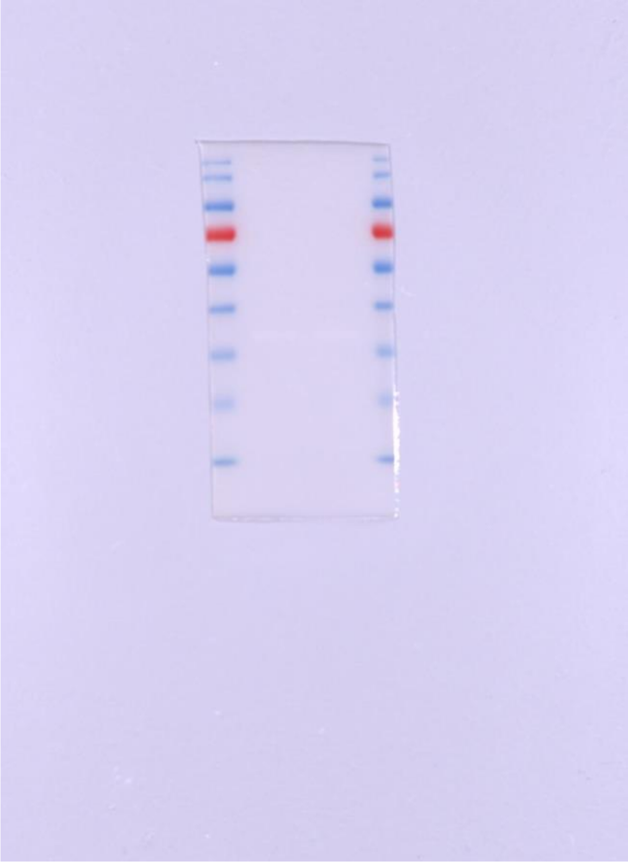

Fig 1E-TU212 ERCC6L

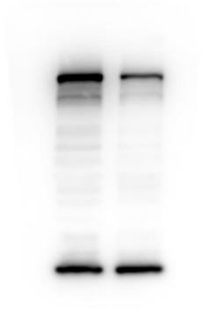

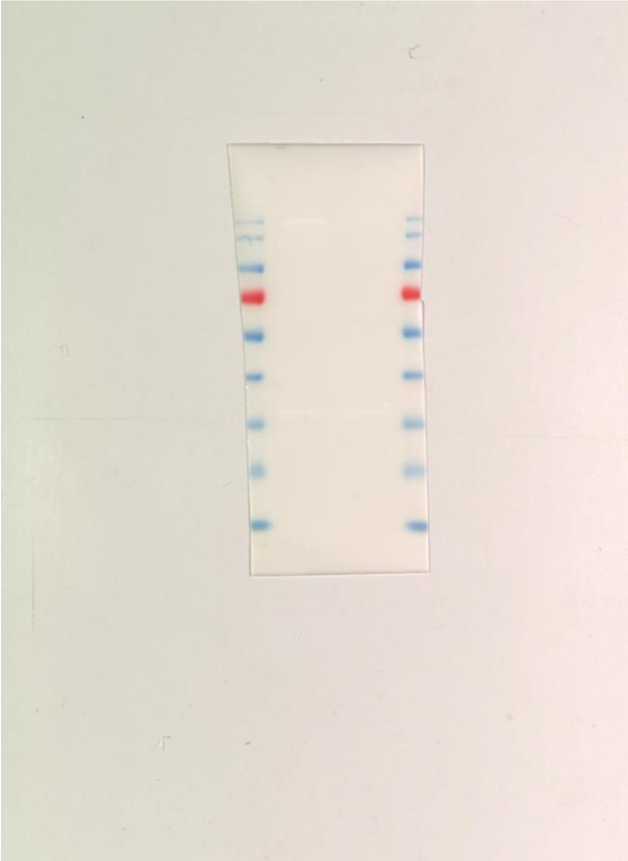

Fig 1E-TU212 GAPDH

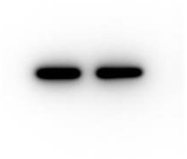

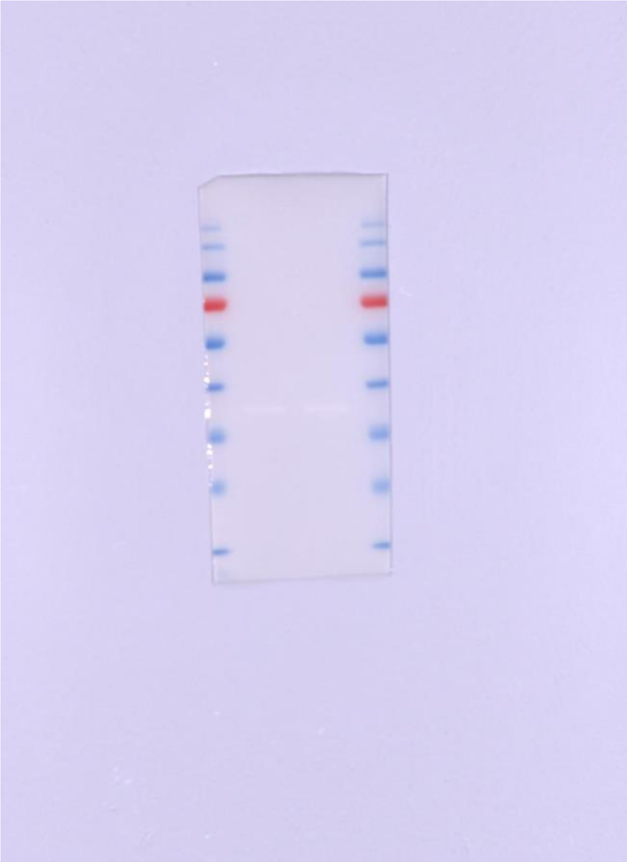

Fig 2G-AMC-HN-8

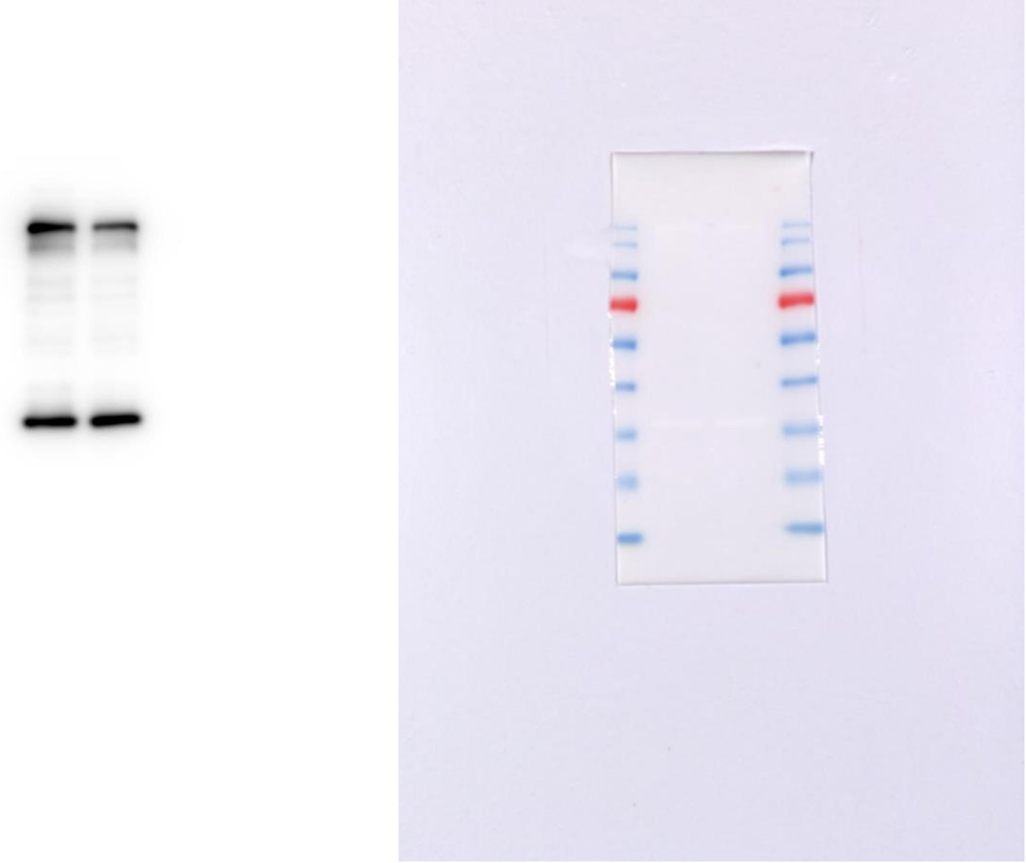

ERCC6L

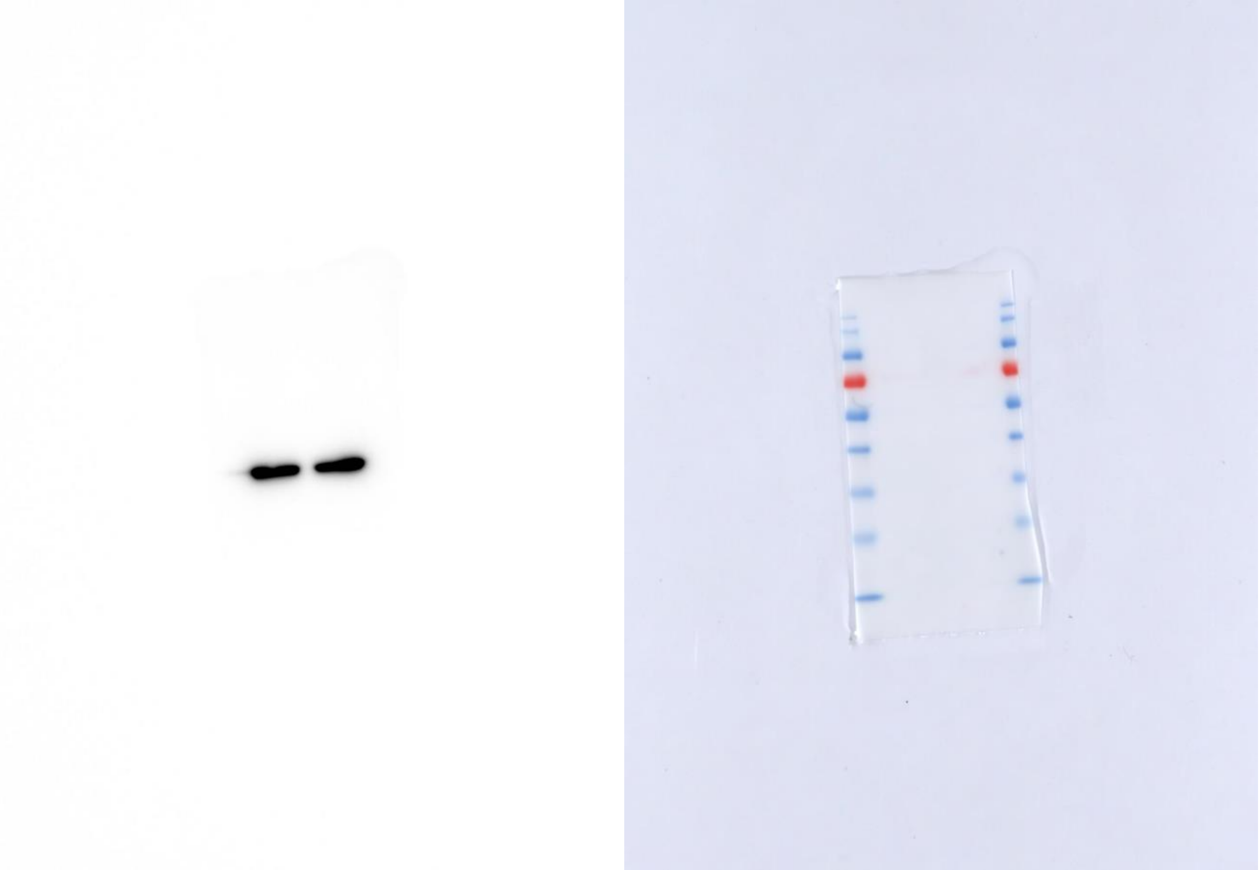

GAPDH

Fig 2G-AMC-HN-8

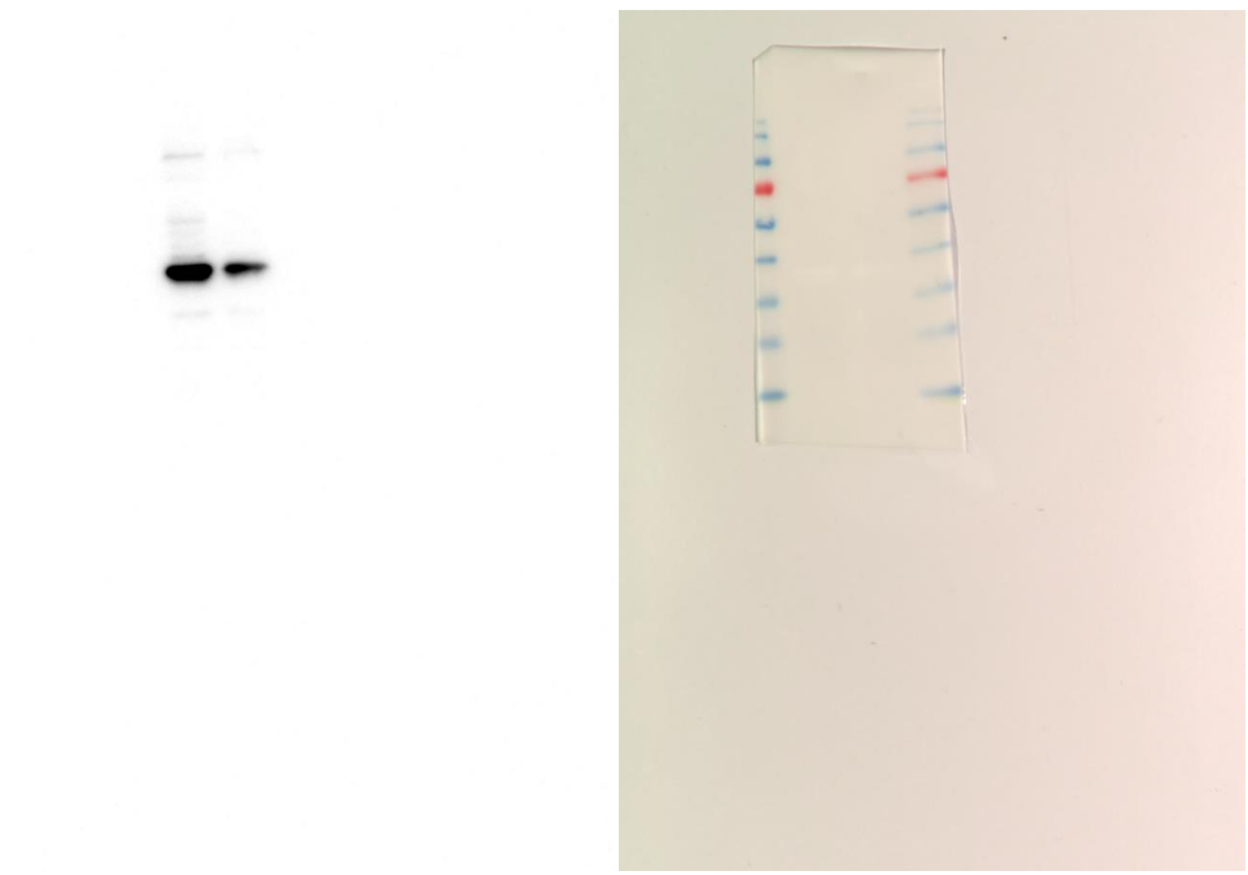

RAD51

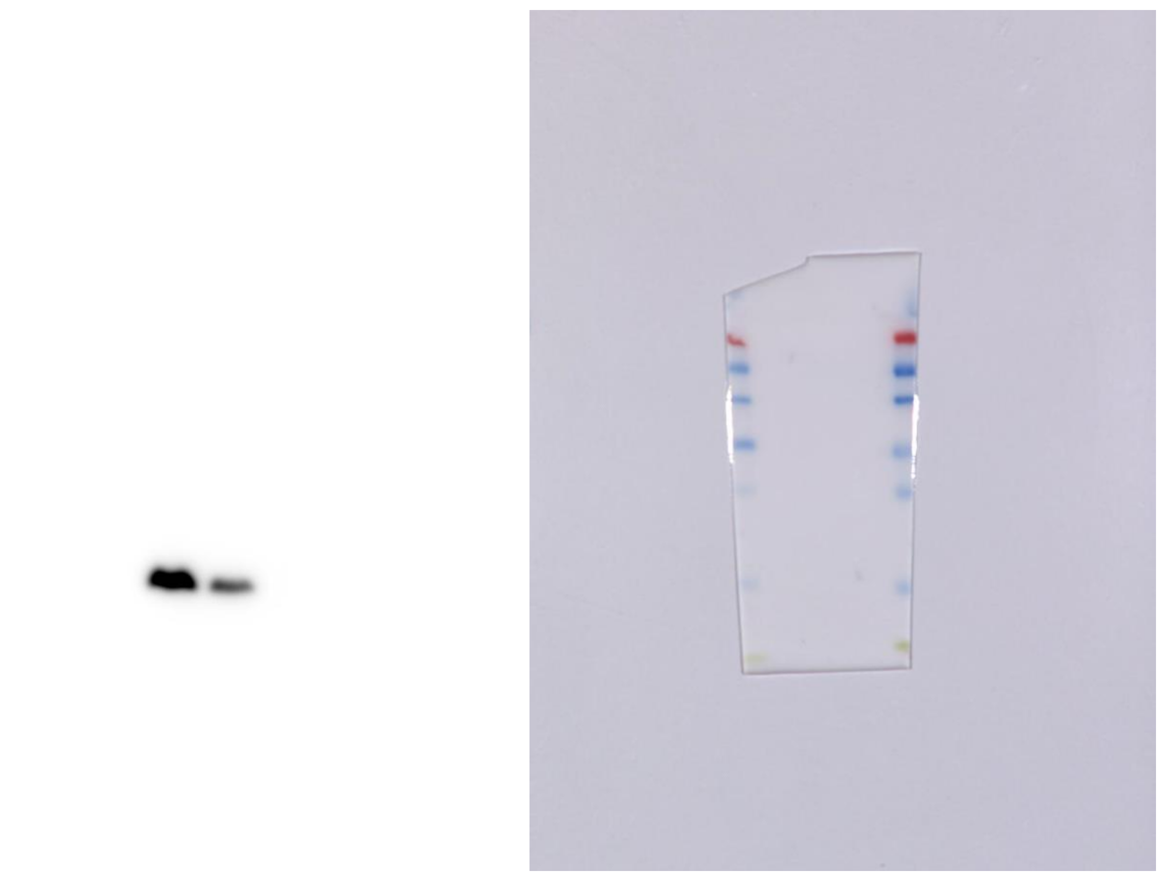

γH2A.X

Fig 2G-TU212

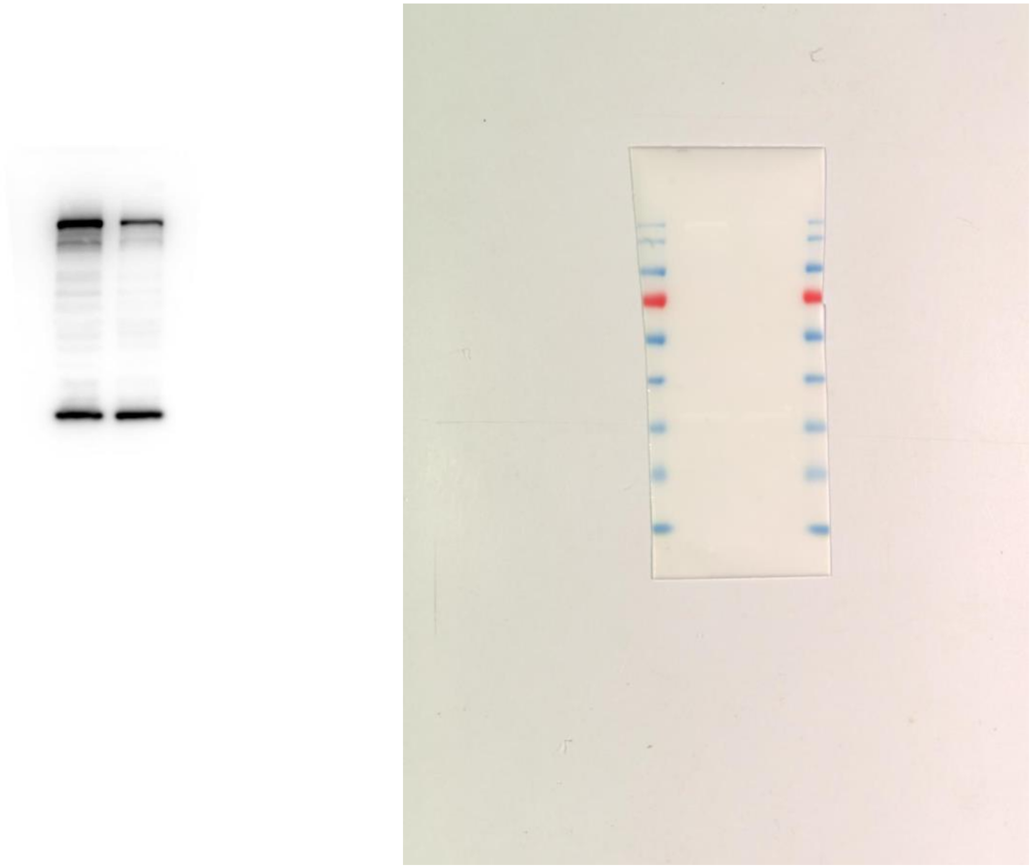

ERCC6L

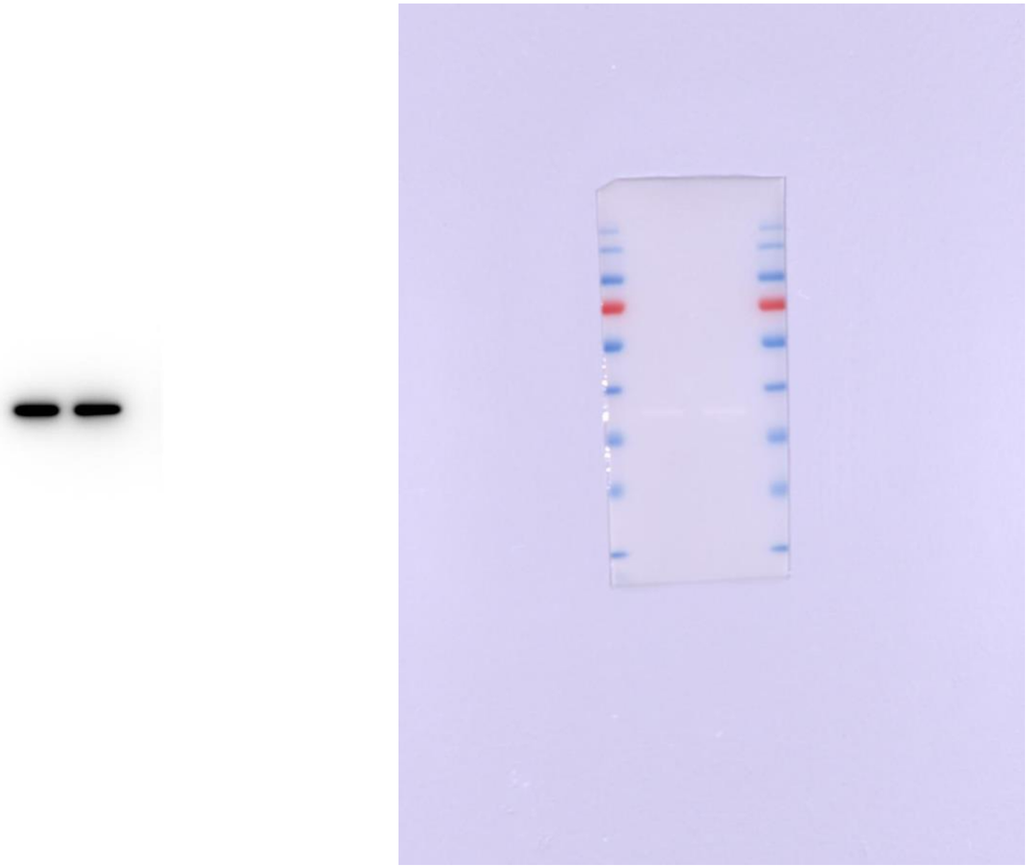

GAPDH

Fig 2G-AMC-HN-8

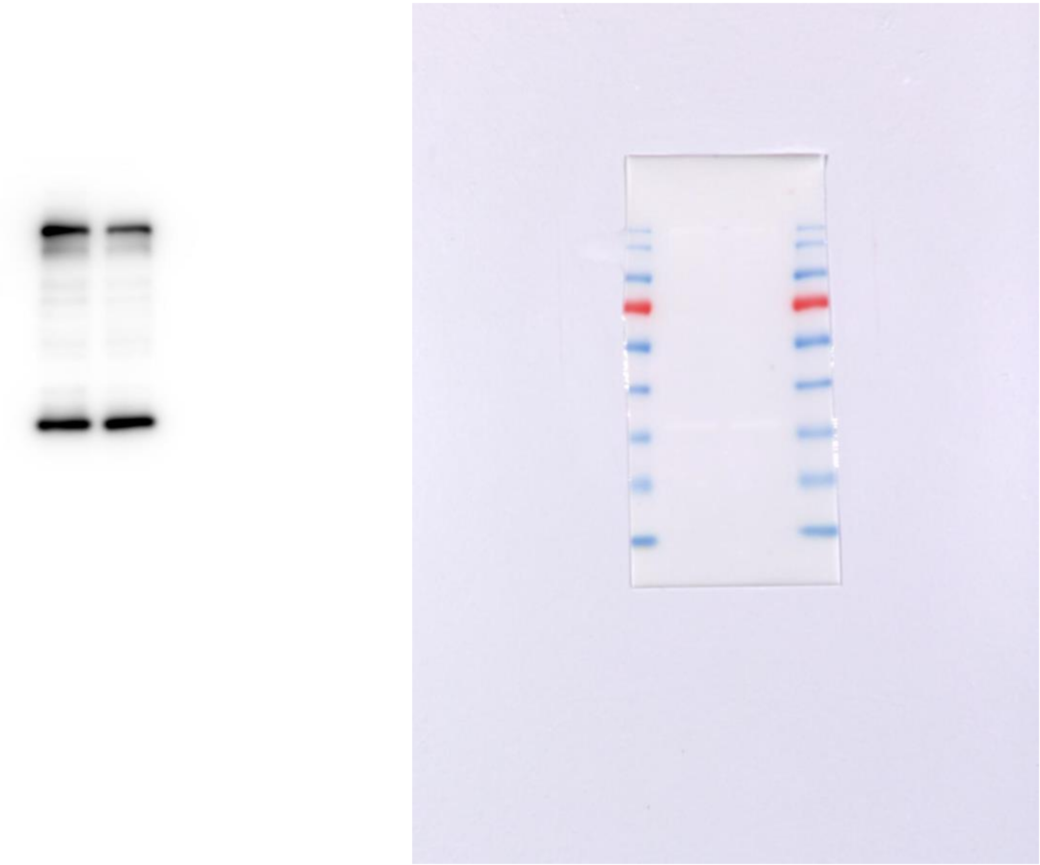

ERCC6L

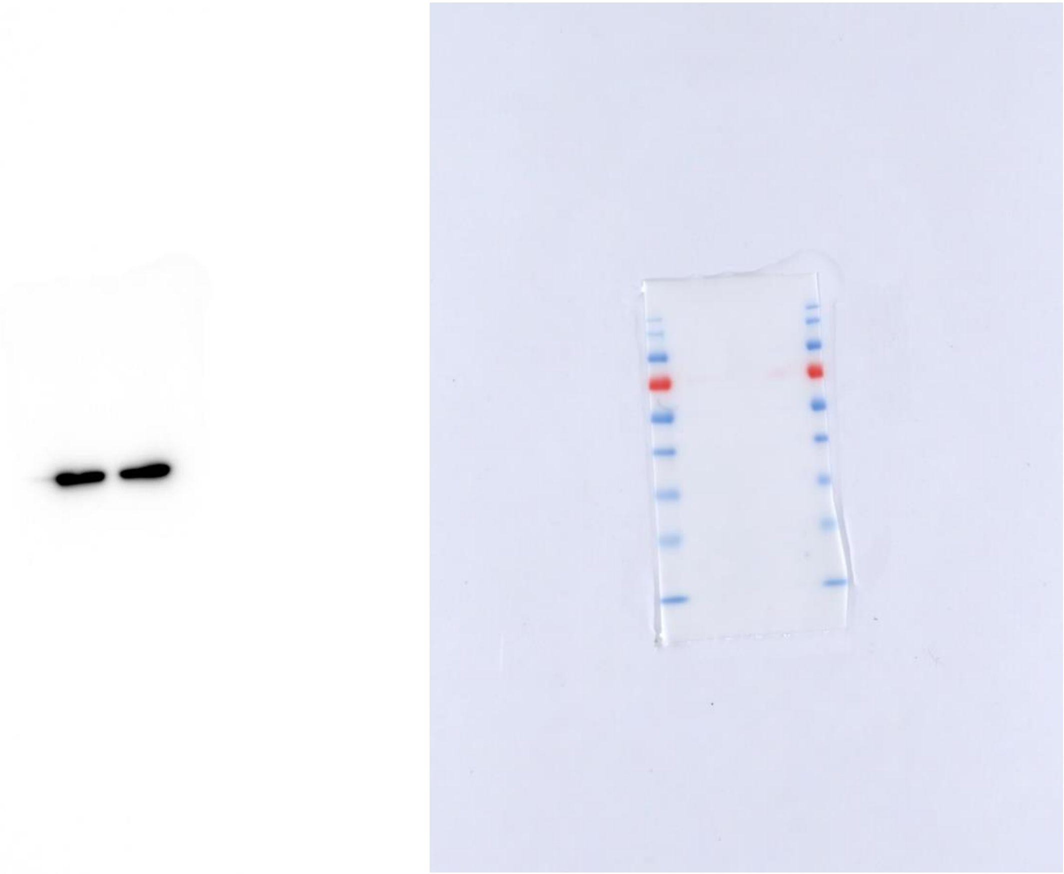

GAPDH

Fig 2G-AMC-HN-8

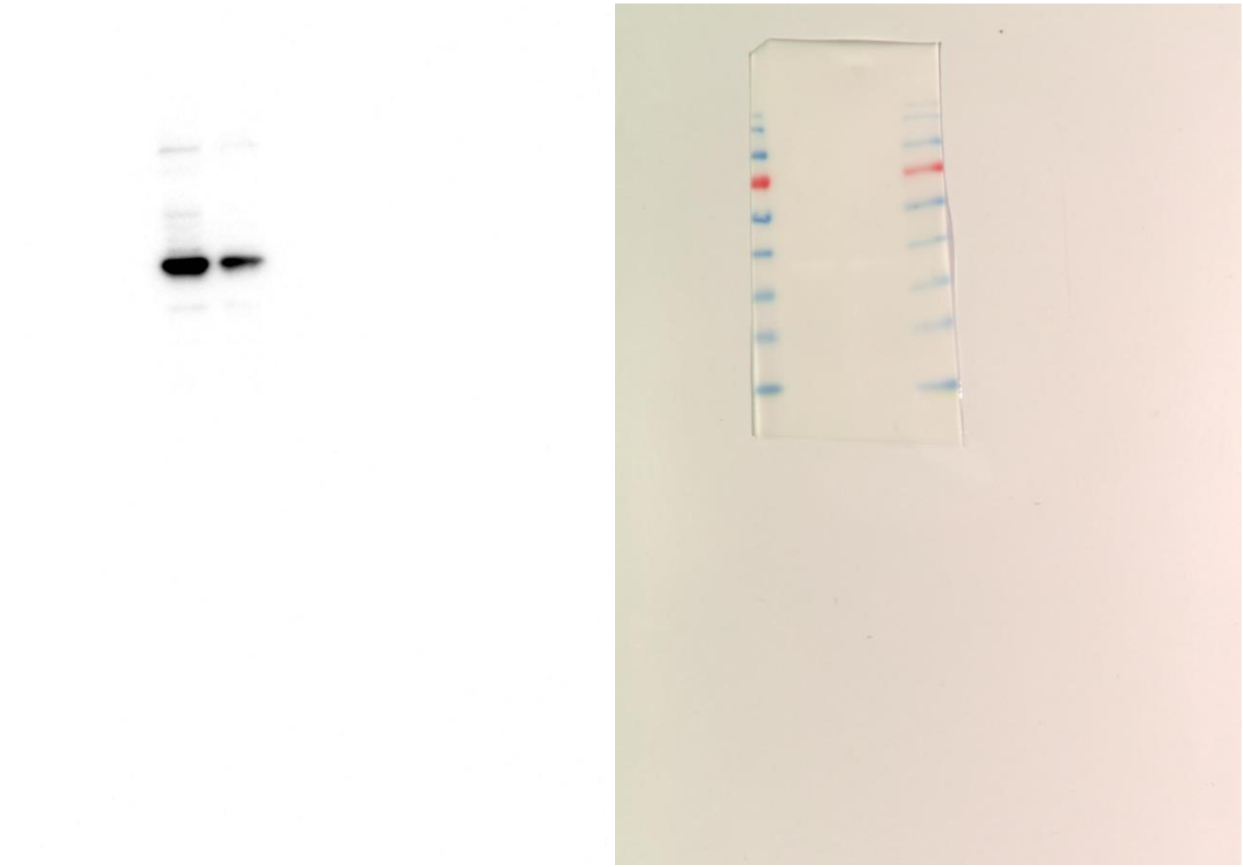

RAD51

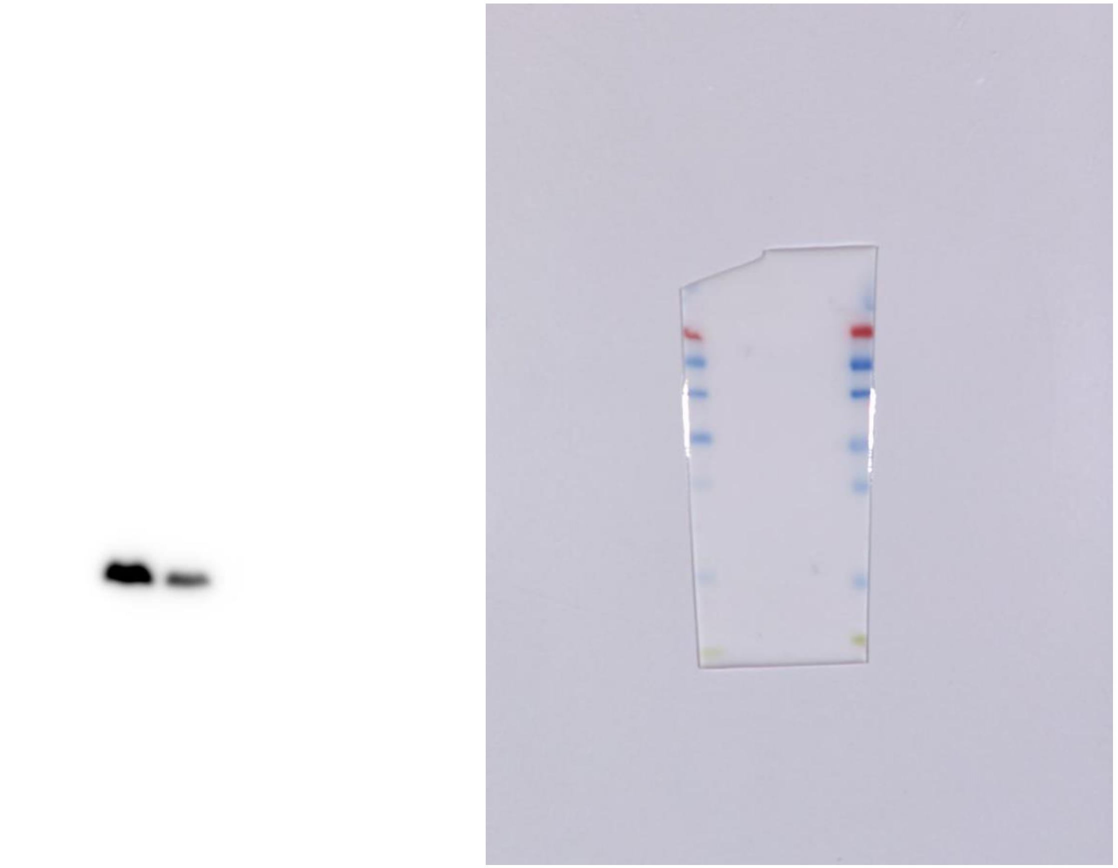

γH2A.X

Fig 2G-TU212

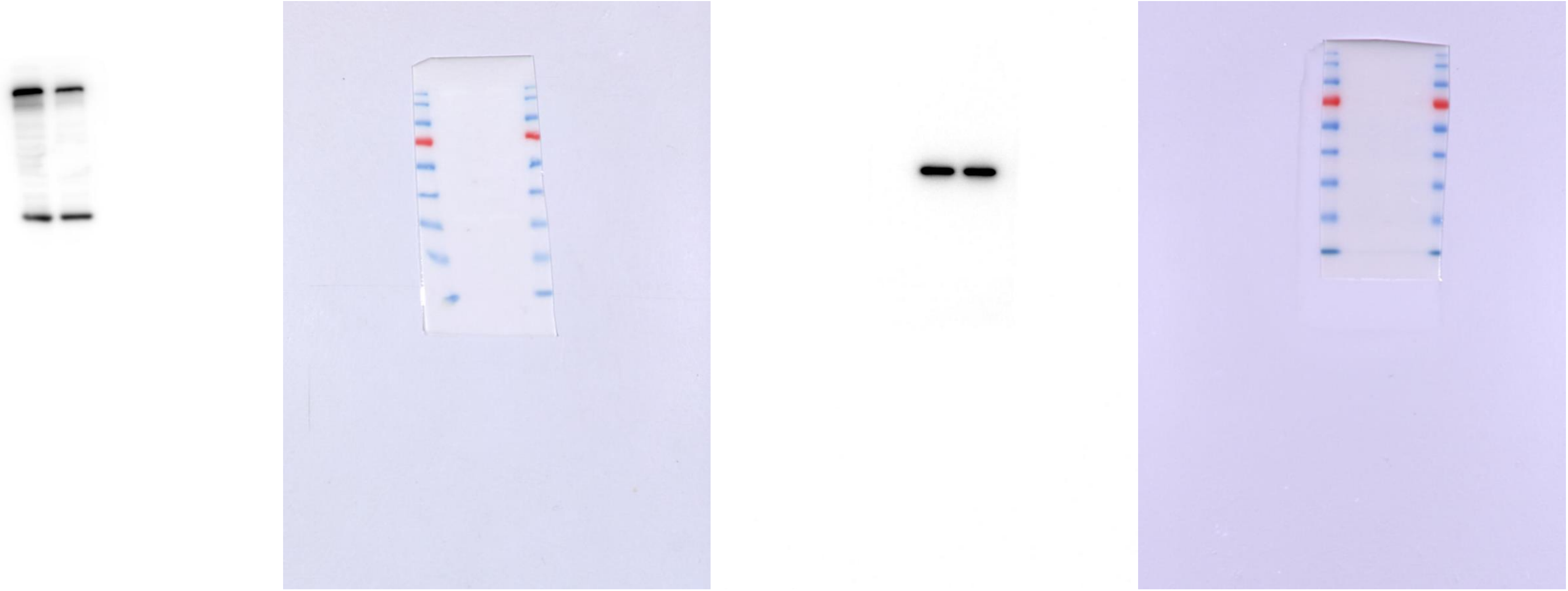

ERCC6L GAPDH

Fig 2G-TU212

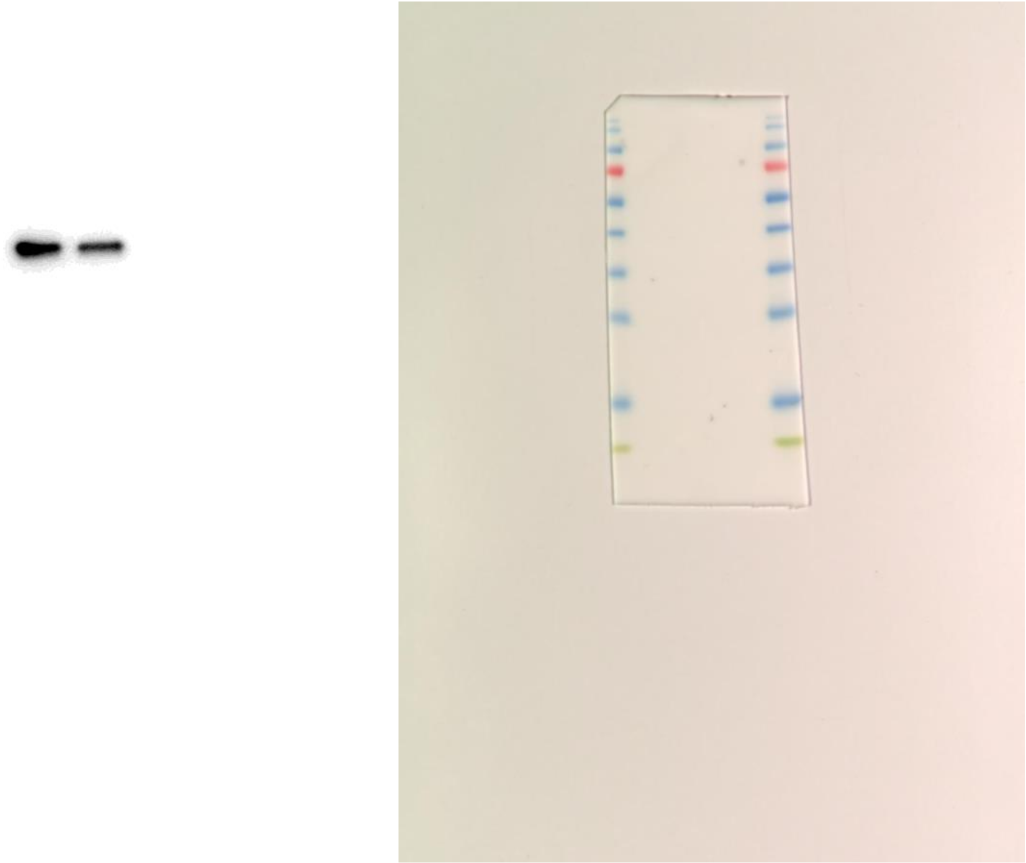

RAD51

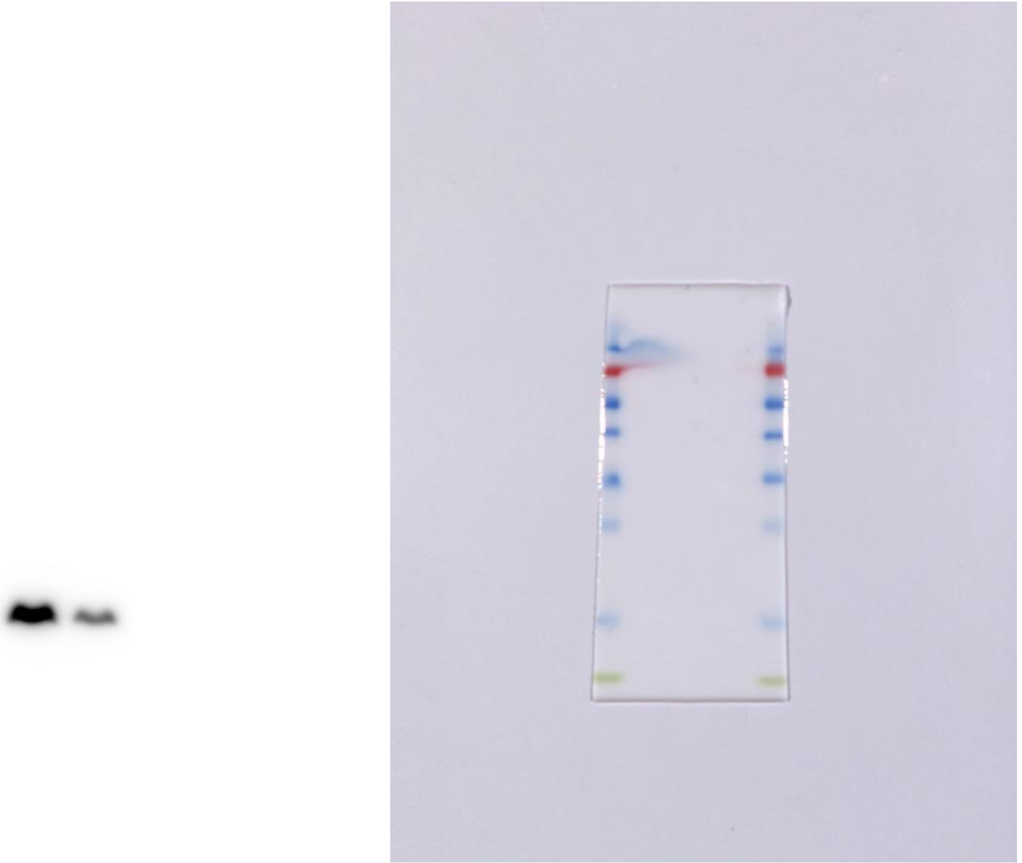

γH2A.X

Fig 3D-AMC-HN-8

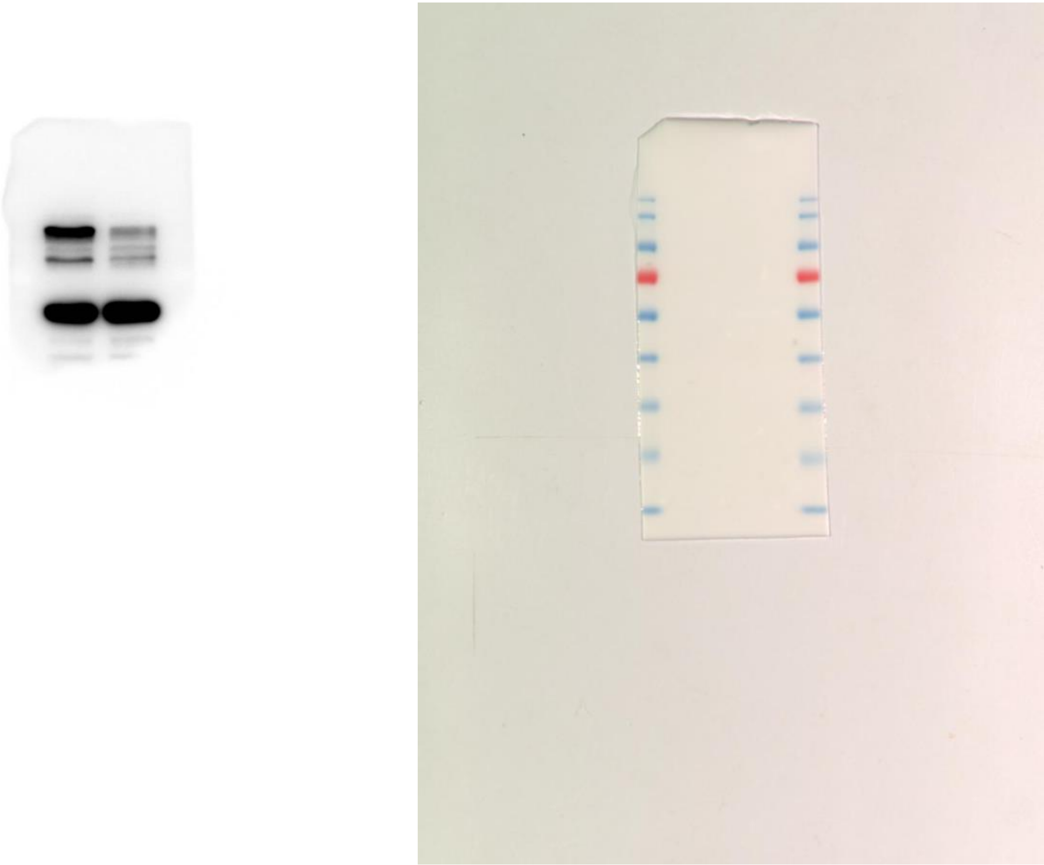

FOXM1

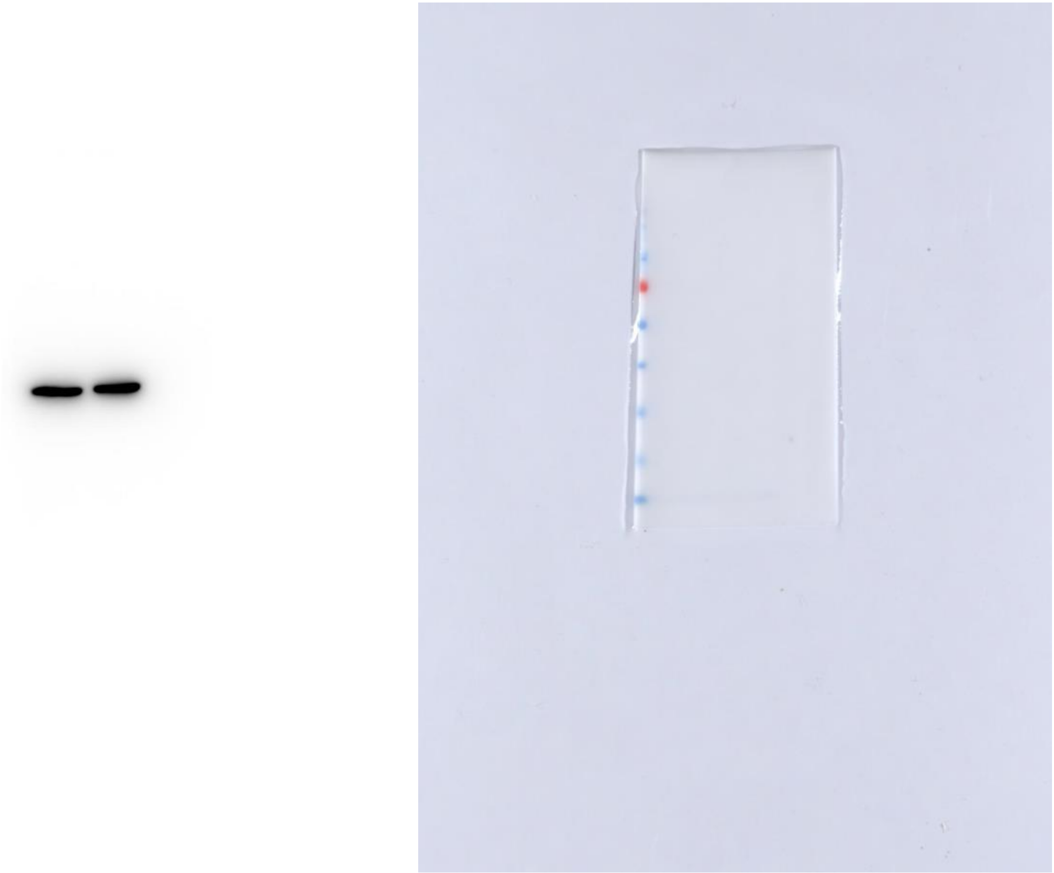

GAPDH

Fig 3D-AMC-HN-8

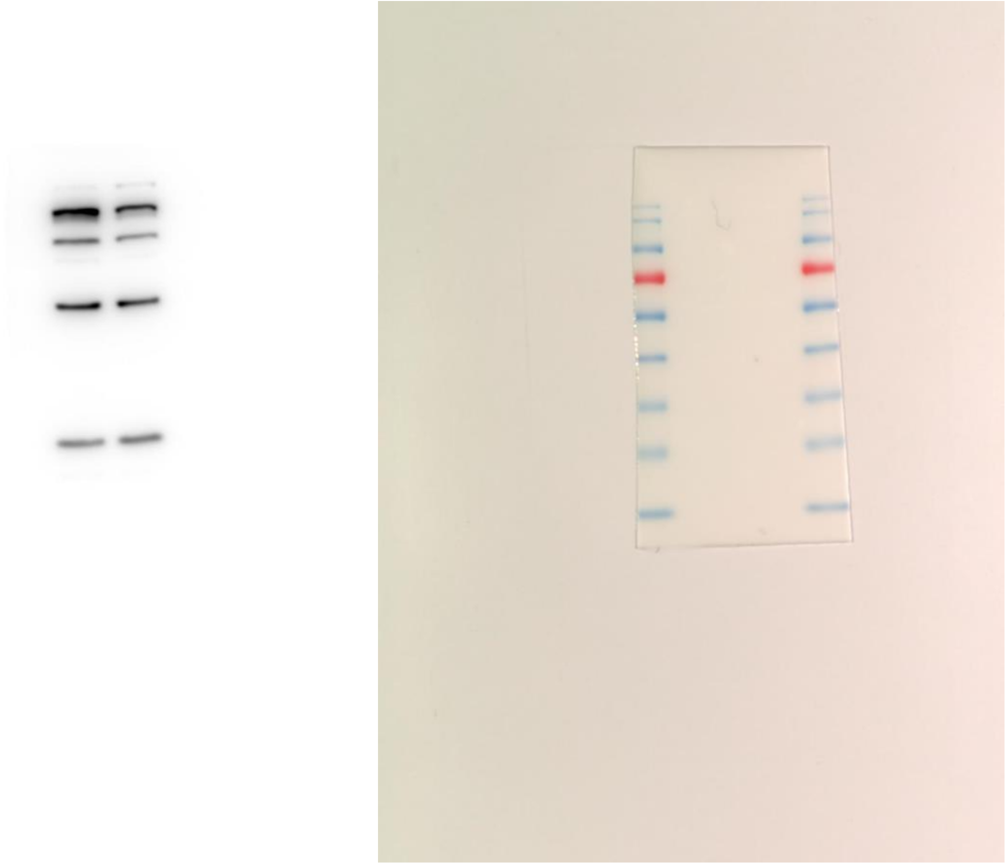

KIF4A

Fig 3D-TU212

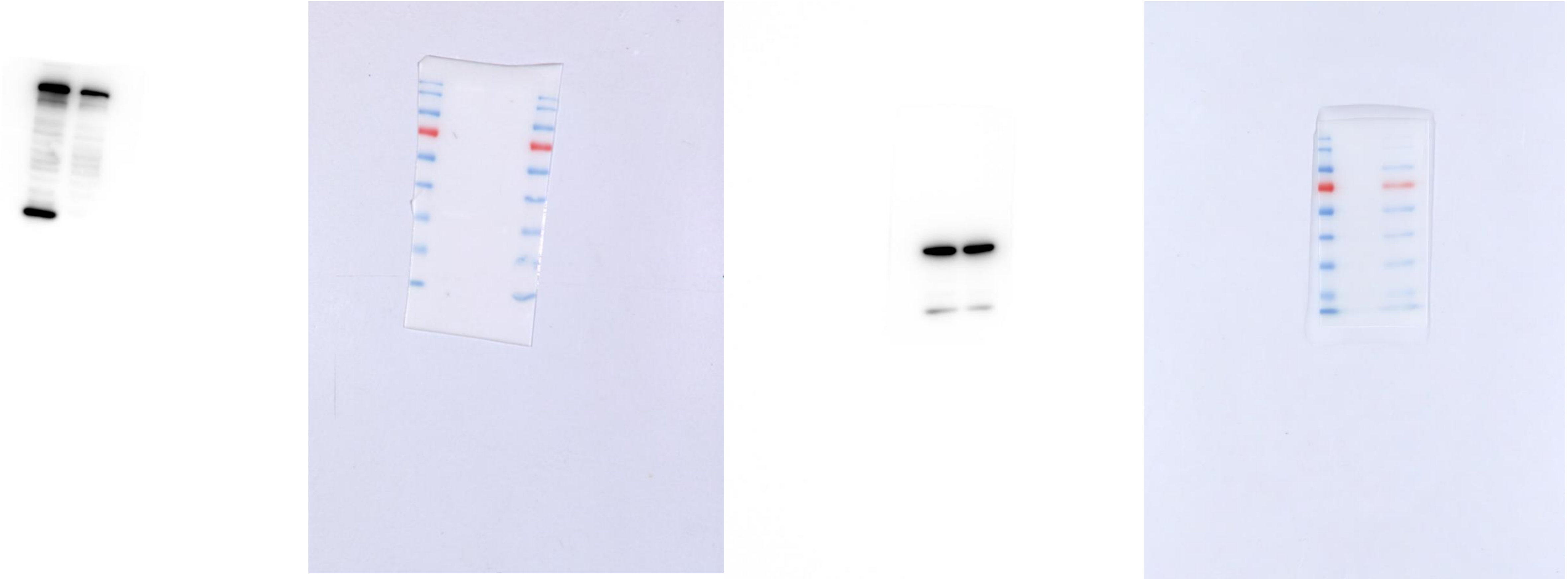

ERCC6L

GAPDH

Fig 3D-TU212

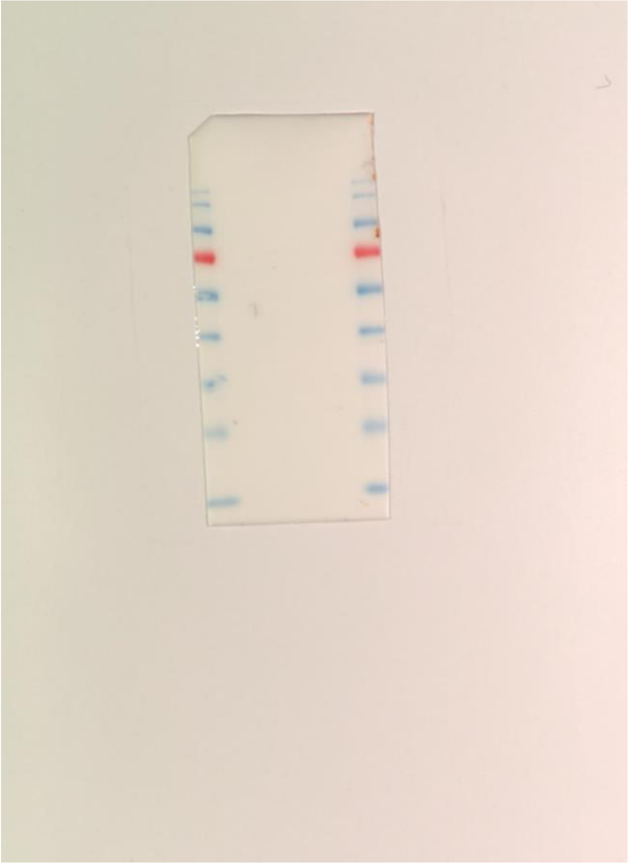

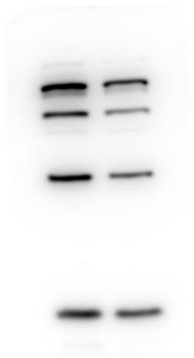

KIF4A

Fig 3F

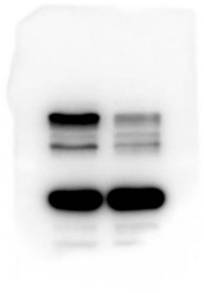
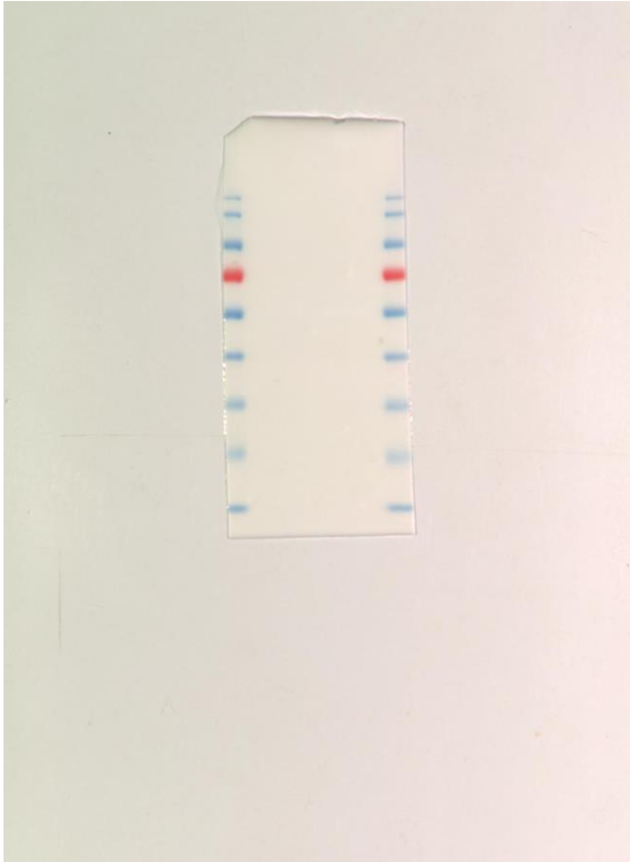

FOXM1

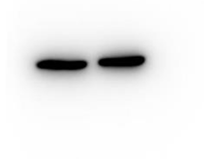
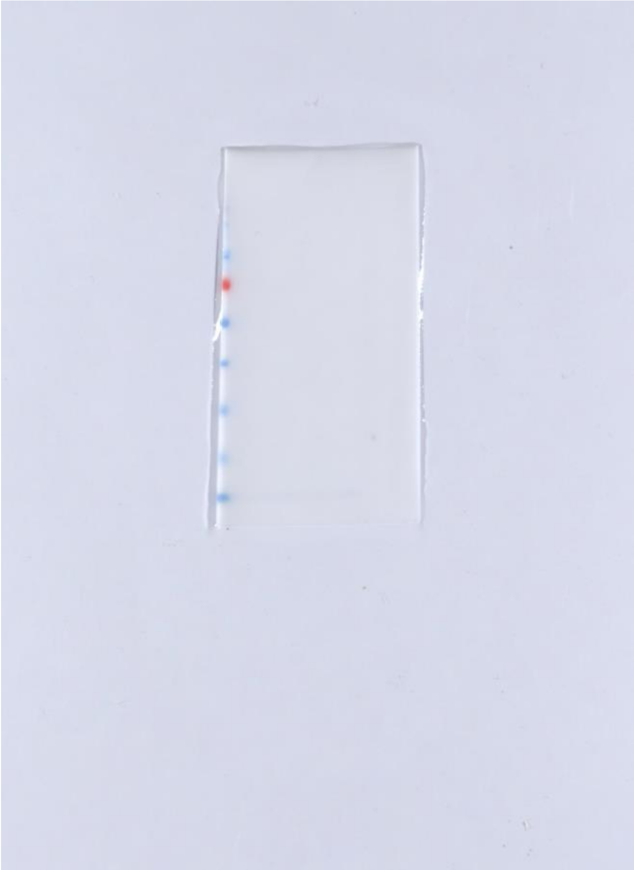

GAPDH

Fig 3F

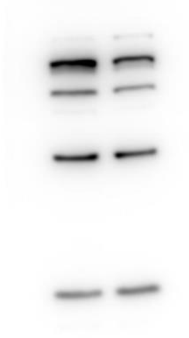

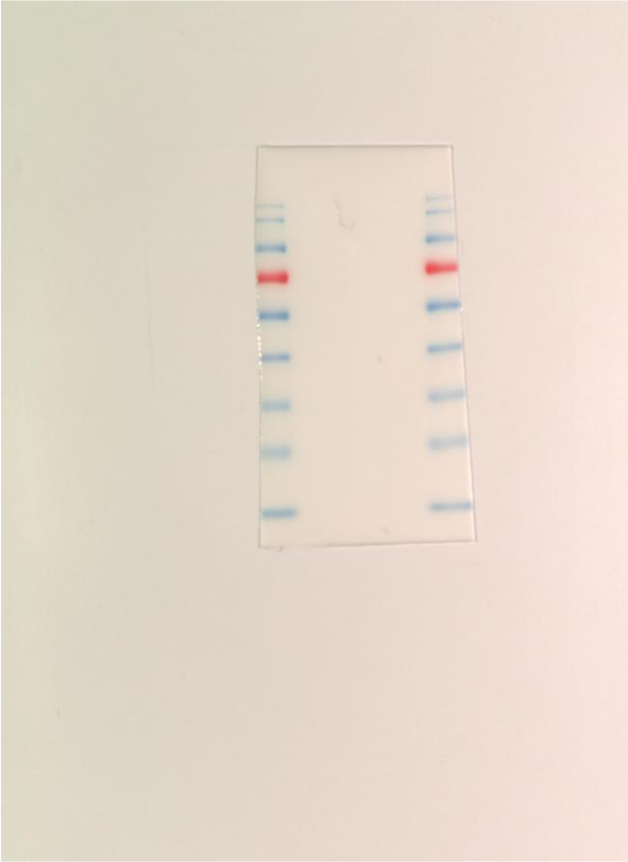

KIF4A
