# Supplementary material for: ERCC6L facilitates the progression of laryngeal squamous cell carcinoma by the binding of FOXM1 and KIF4A
Source: Cell Death Discov. 2023 Feb 2;9:41. doi: 10.1038/s41420-023-01314-3 (PMC9892579; doi:10.1038/s41420-023-01314-3)
Supplement: Supplementary file 2 — Original Data File [file 41420_2023_1314_MOESM2_ESM.pdf]

一、Raw data

Group	Number	Picture number	ROI	Total fluorescent expression in the region[p/s] / [μW/cm²]	Every cm in the area ² Average fluorescent expression[p/s/cm²/sr] / [μW/cm²]	Standard deviation of fluorescent expression	Minimum fluorescent expression in the region	Maximum fluorescent expression in the region
OE+NC(KD)	1	CLS20220207150813_001A	ROI 1	5.11E+11	6.57E+09	1.42E+10	1.88E+07	1.05E+11
	2	CLS20220207150813_001A	ROI 2	1.07E+12	1.37E+10	2.19E+10	4.49E+07	1.03E+11
	3	CLS20220207150813_001A	ROI 3	1.16E+12	1.50E+10	2.27E+10	6.27E+07	1.15E+11
	4	CLS20220207150955_001A	ROI 4	3.78E+11	4.86E+09	8.57E+09	5.07E+06	4.92E+10
NC(OE+KD)	5	CLS20220207151055_001A	ROI 5	1.52E+12	1.95E+10	2.70E+10	5.22E+07	1.02E+11
	6	CLS20220207151055_001A	ROI 6	9.09E+11	1.17E+10	1.80E+10	6.82E+07	9.23E+10
	7	CLS20220207151055_001A	ROI 7	5.12E+11	6.58E+09	1.08E+10	3.11E+07	6.95E+10
	8	CLS20220207151144_001A	ROI 8	7.17E+11	9.23E+09	1.49E+10	1.95E+07	7.14E+10
OE+KD	9	CLS20220207151249_001A	ROI 1	4.89E+11	6.28E+09	1.42E+10	5.27E+06	7.67E+10
	10	CLS20220207151249_001A	ROI 2	1.07E+12	1.38E+10	2.40E+10	5.21E+07	1.07E+11
	11	CLS20220207151249_001A	ROI 3	9.45E+11	1.22E+10	2.33E+10	3.96E+07	1.12E+11
	12	CLS20220207151339_001A	ROI 4	7.51E+11	9.65E+09	1.68E+10	1.82E+07	7.65E+10
KD+NC(OE)	13	CLS20220207151437_001A	ROI 5	7.83E+11	1.01E+10	2.04E+10	5.14E+06	8.76E+10
	14	CLS20220207151437_001A	ROI 6	6.01E+11	7.73E+09	1.36E+10	2.98E+07	8.08E+10
	15	CLS20220207151437_001A	ROI 7	2.09E+11	2.69E+09	7.70E+09	1.34E+07	5.38E+10
	16	CLS20220207151526_001A	ROI 8	1.98E+11	2.55E+09	7.29E+09	8.83E+06	4.43E+10

二、Data processing

AVERAGE_Total fluorescent expression in the region ([p/s] / [μW/cm²])

AVERAGE_Total fluorescent expression in the region ([p/s] / [μW/cm²])	Group			
	OE+NC(KD)	NC(OE+KD)	OE+KD	KD+NC(OE)
	7.80E+11	9.14E+11	8.15E+11	4.48E+11

T-TEST:

T-TEST_Total fluorescent expression in the region			P VALUE
OE+NC(KD)	VS	NC(OE+KD)	0.66335151
OE+NC(KD)	VS	OE+KD	0.88638466
NC(OE+KD)	VS	KD+NC(OE)	0.70742761
OE+KD	VS	KD+NC(OE)	0.12510995

STDEV_Total fluorescent expression in the region ([p/s] / [μW/cm²])

STDEV_Total fluorescent expression in the region ([p/s] / [μ	Group			
	OE+NC(KD)	NC(OE+KD)	OE+KD	KD+NC(OE)
	3.93E+11	4.34E+11	4.79E+09	6.14E+09
