# Supplementary figures and images for: ERCC6L facilitates the progression of laryngeal squamous cell carcinoma by the binding of FOXM1 and KIF4A
Source: Cell Death Discov. 2023 Feb 2;9:41. doi: 10.1038/s41420-023-01314-3 (PMC9892579; doi:10.1038/s41420-023-01314-3)

NC

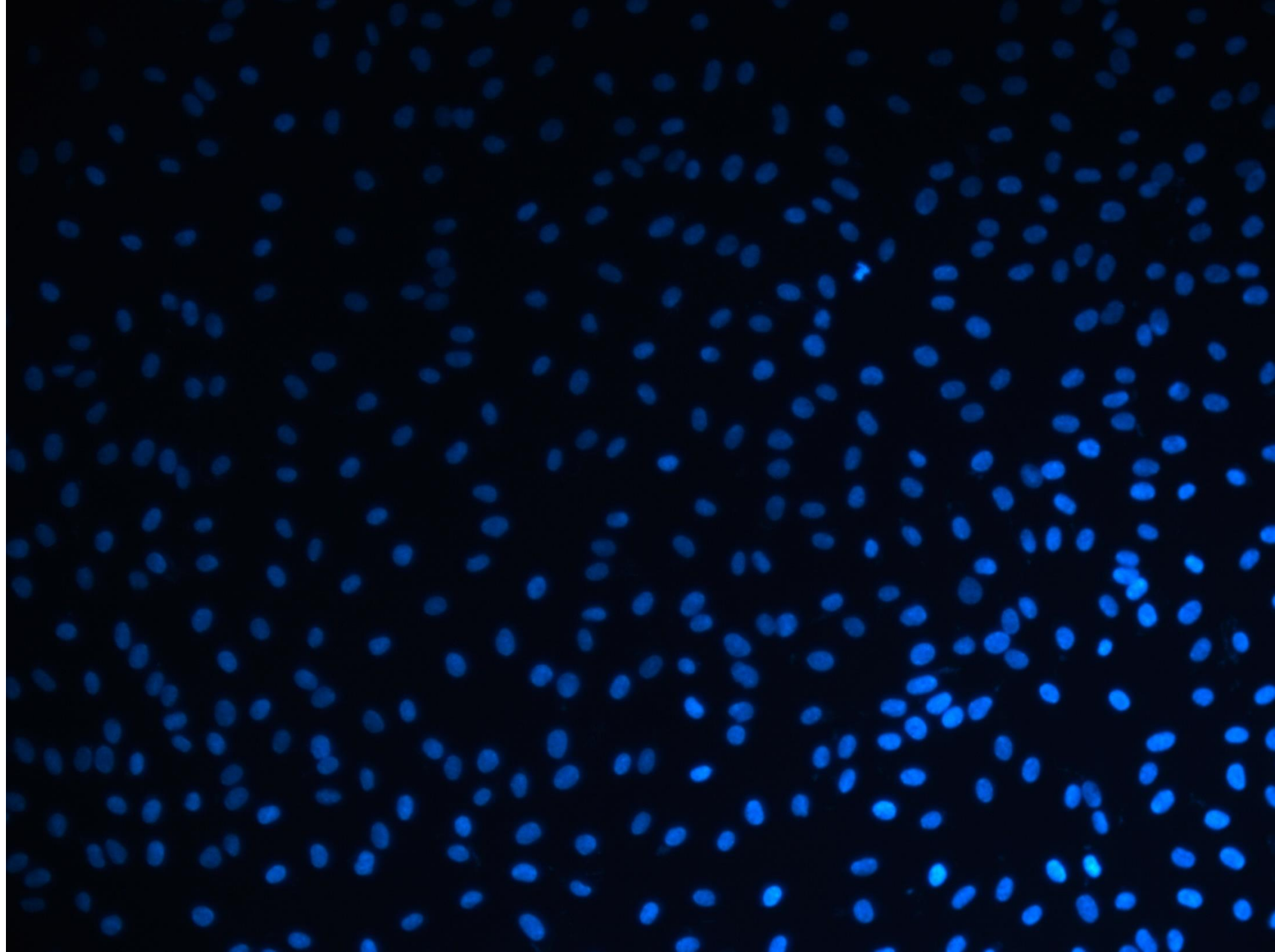

NC

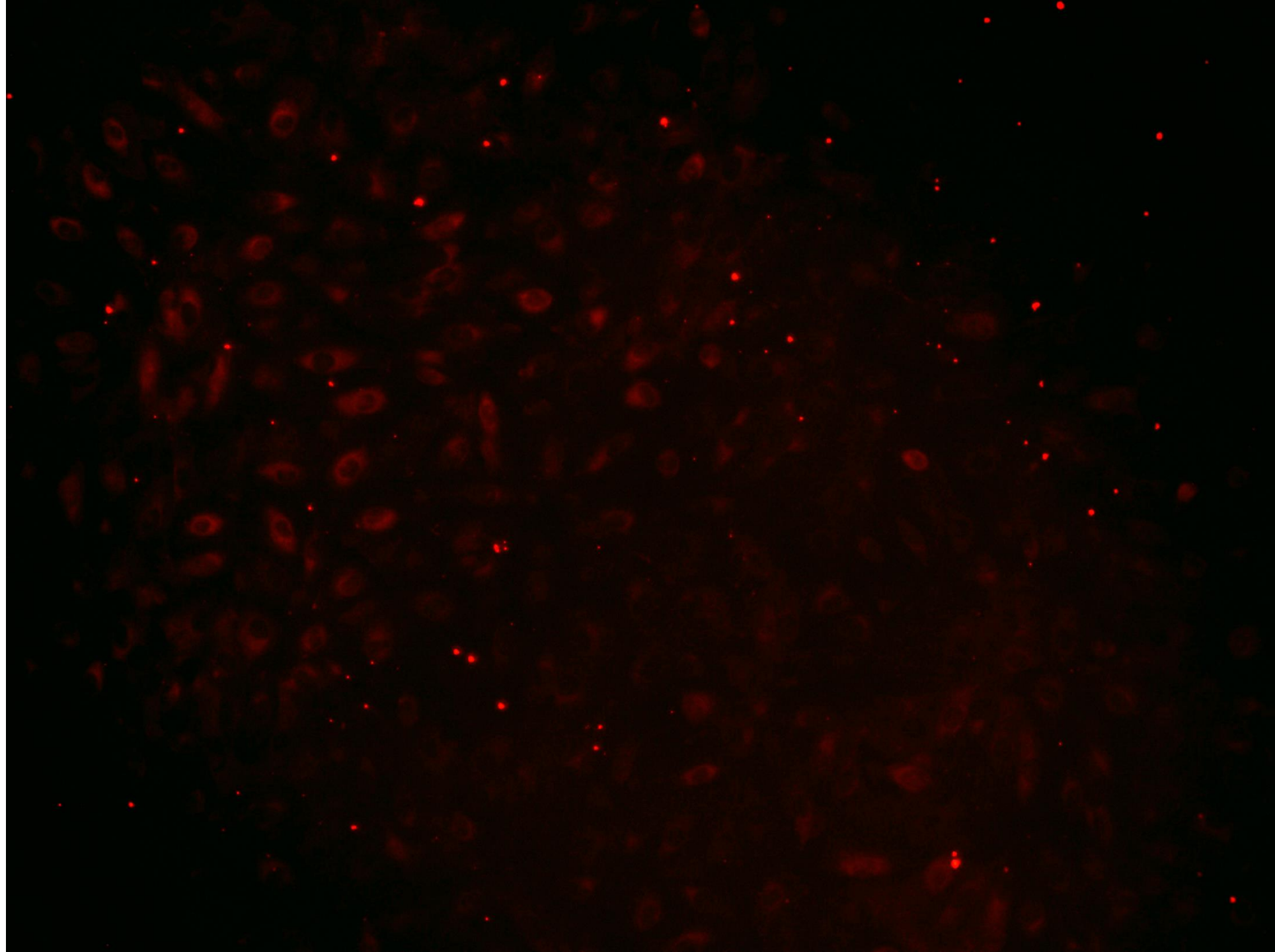

NC

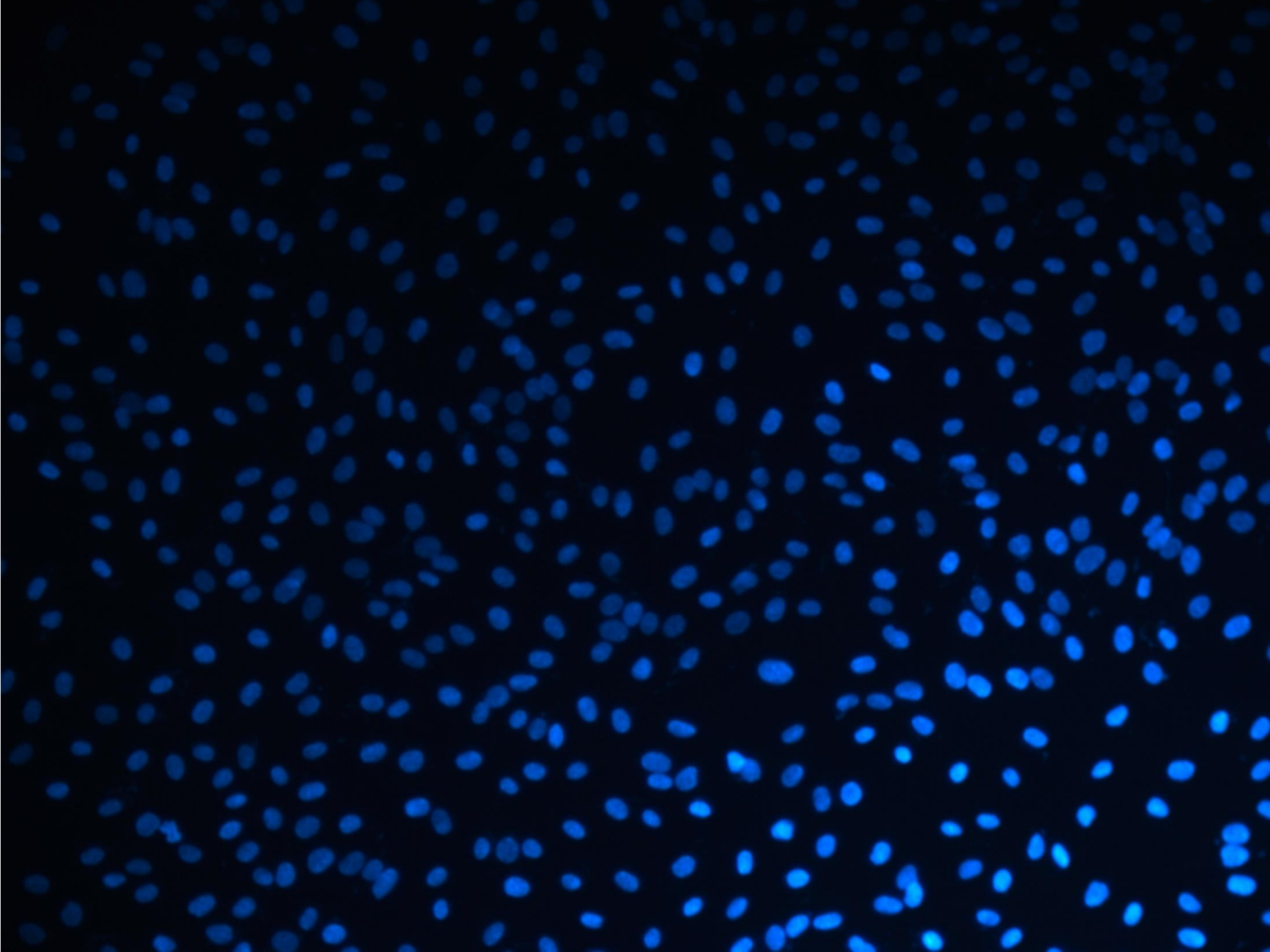

NC

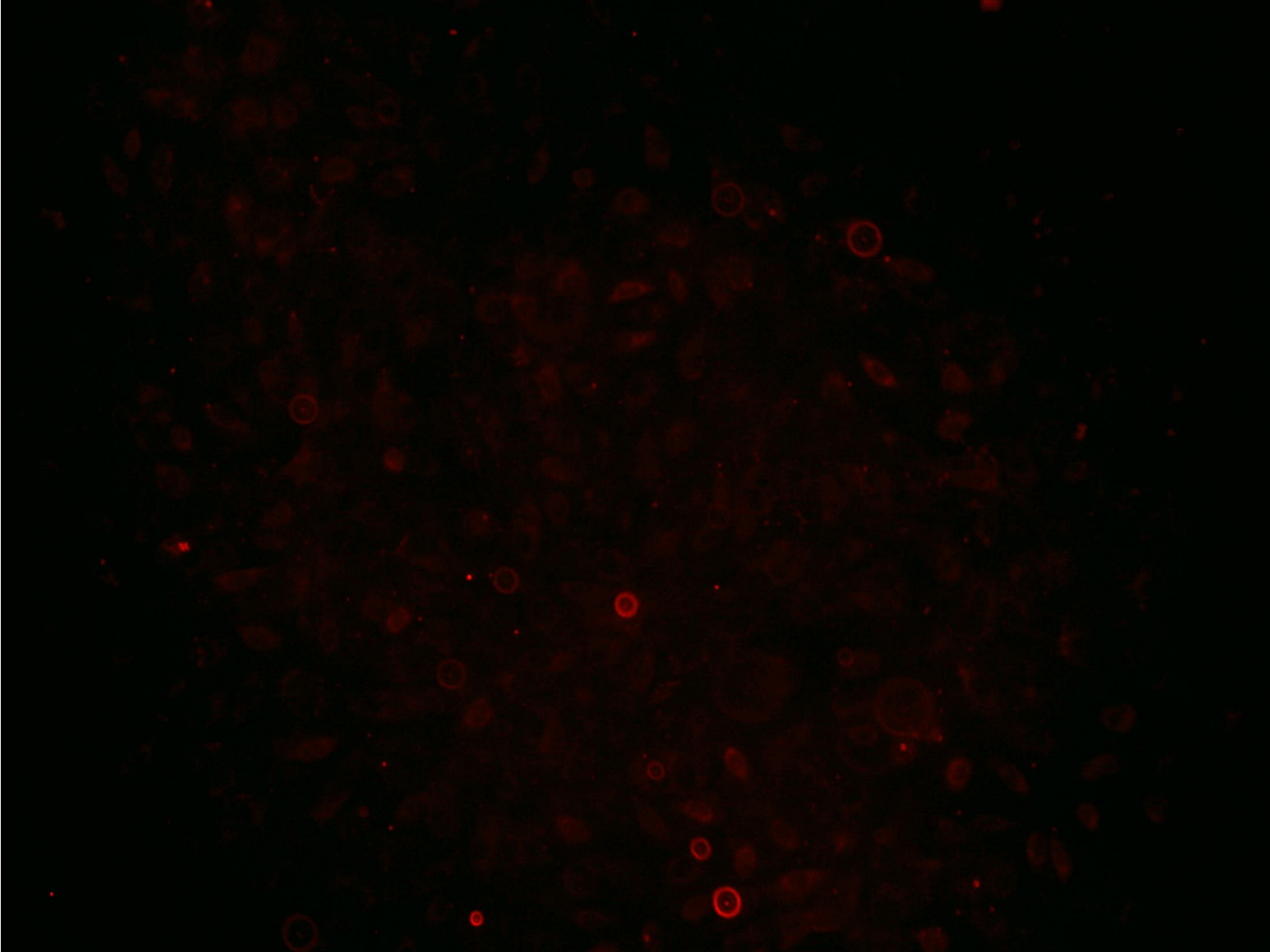

NC

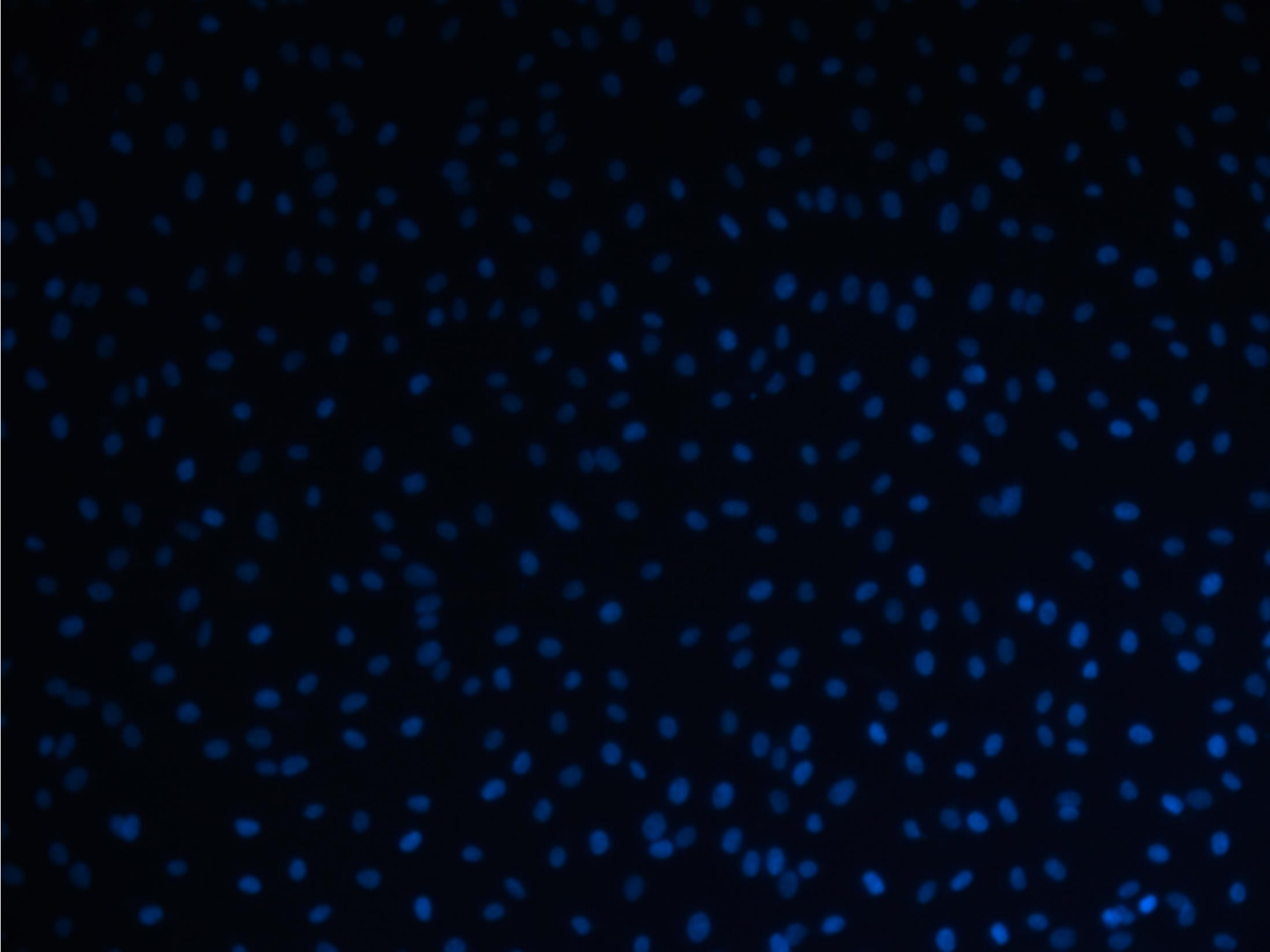

NC

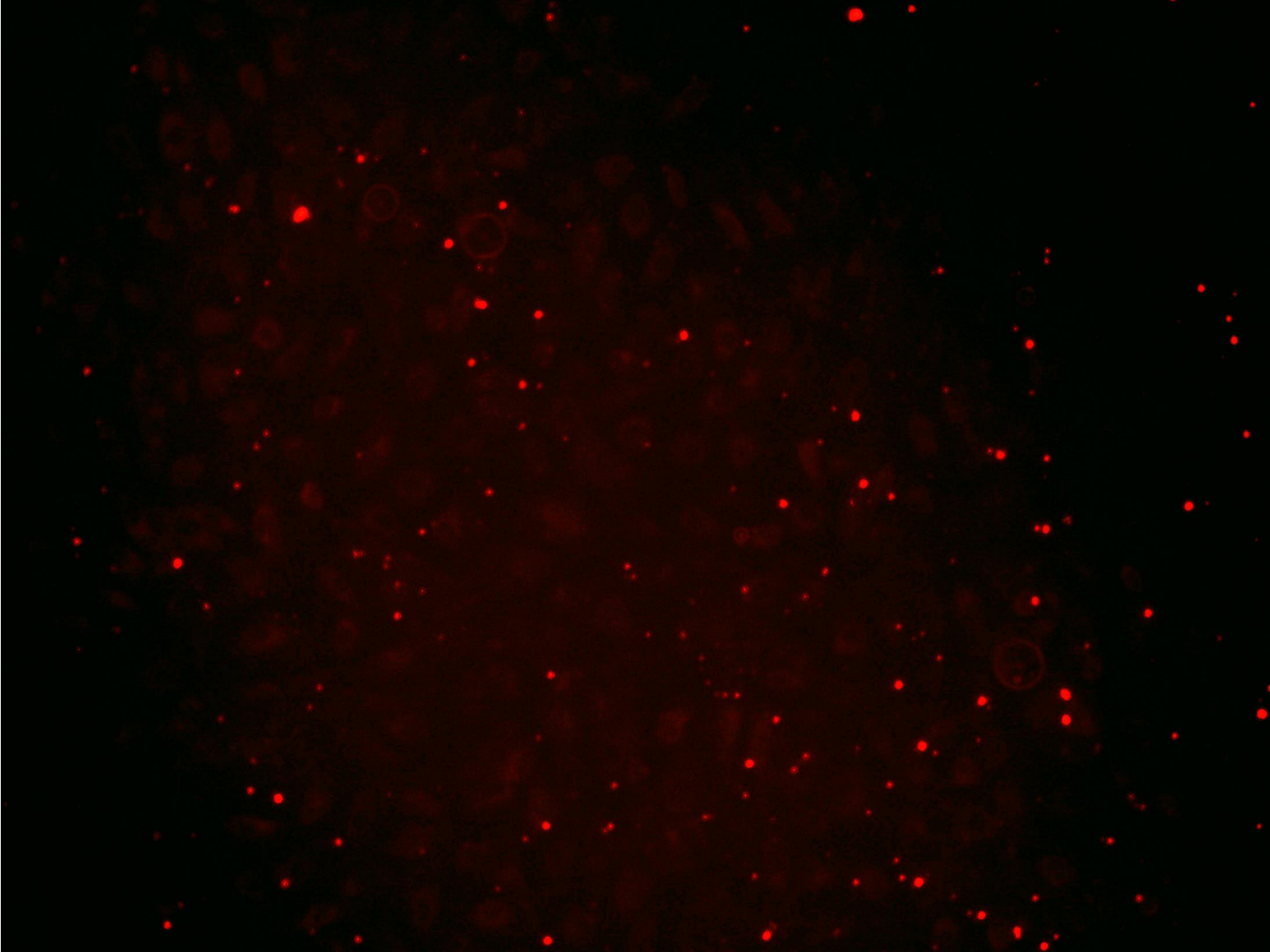

OE-ERCC6L

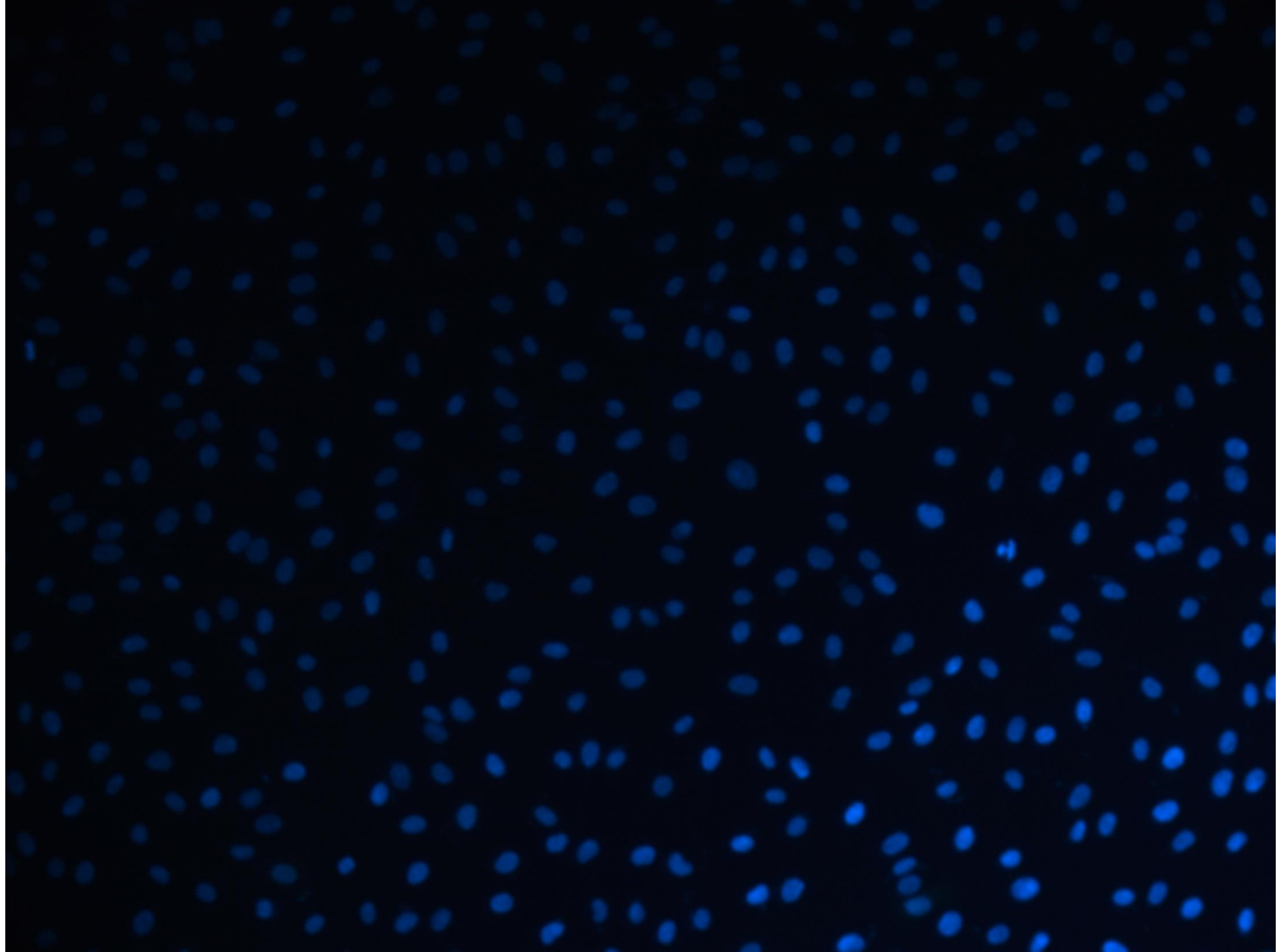

OE-ERCC6L

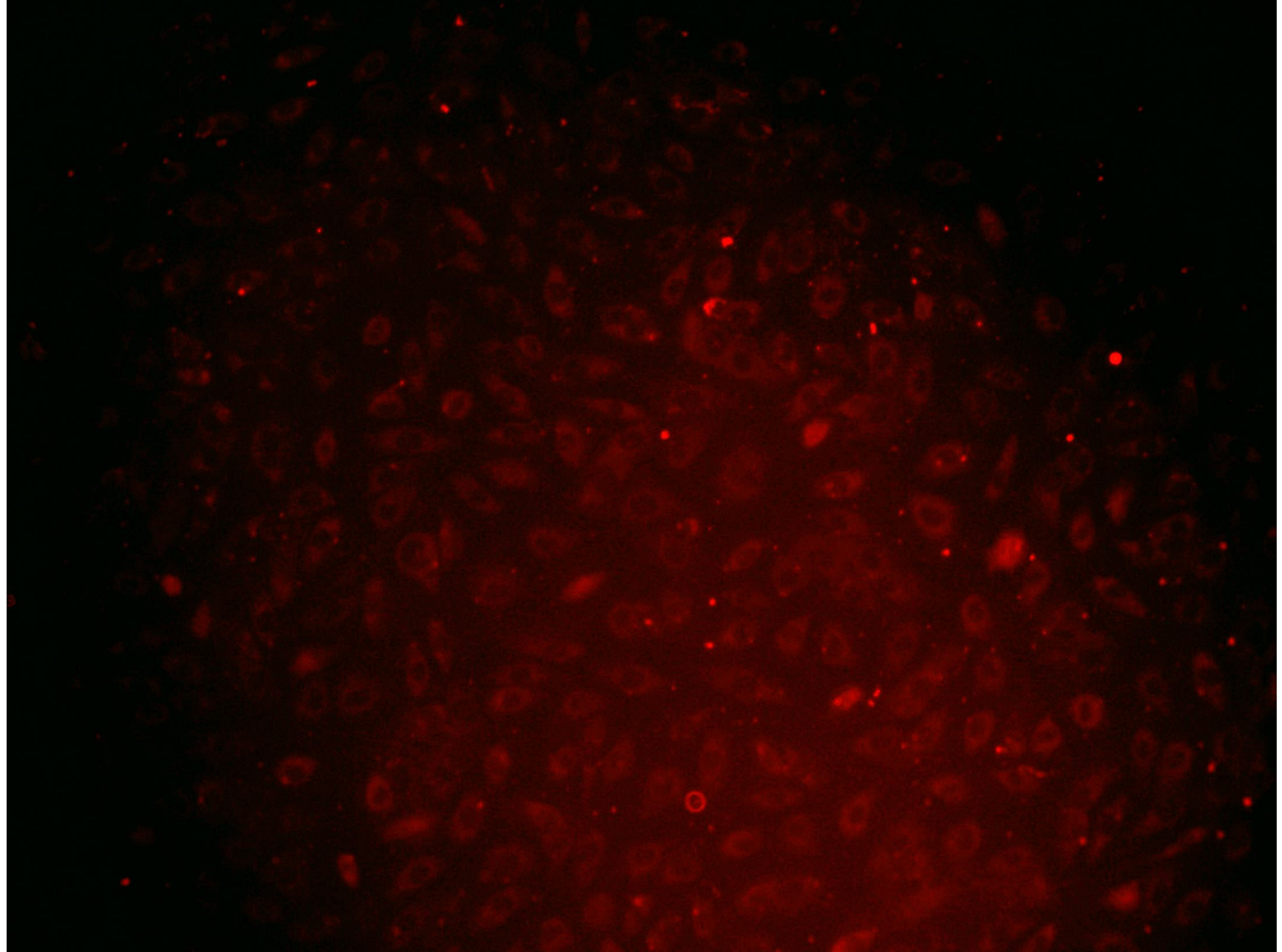

OE-ERCC6L

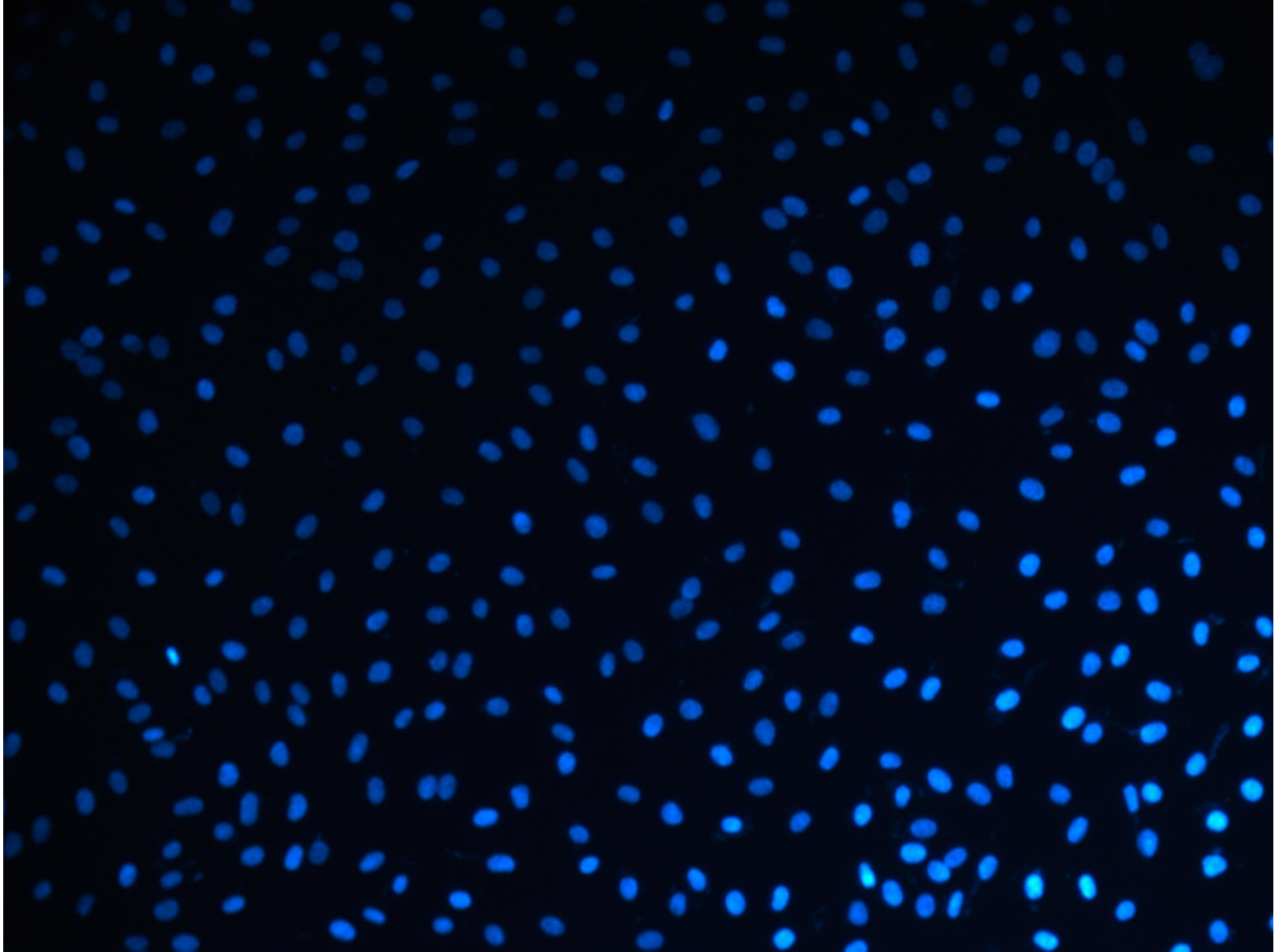

OE-ERCC6L

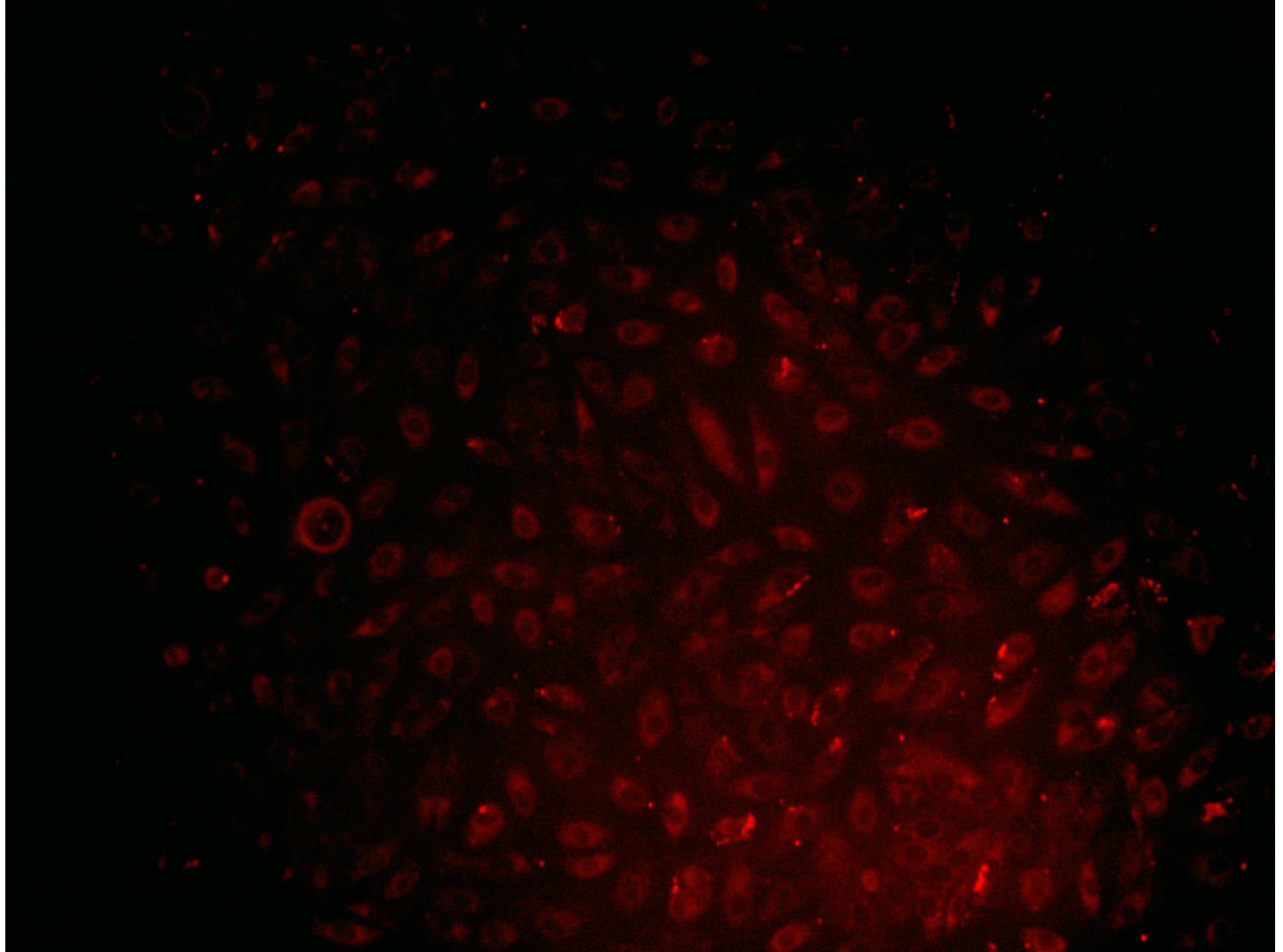

OE-ERCC6L

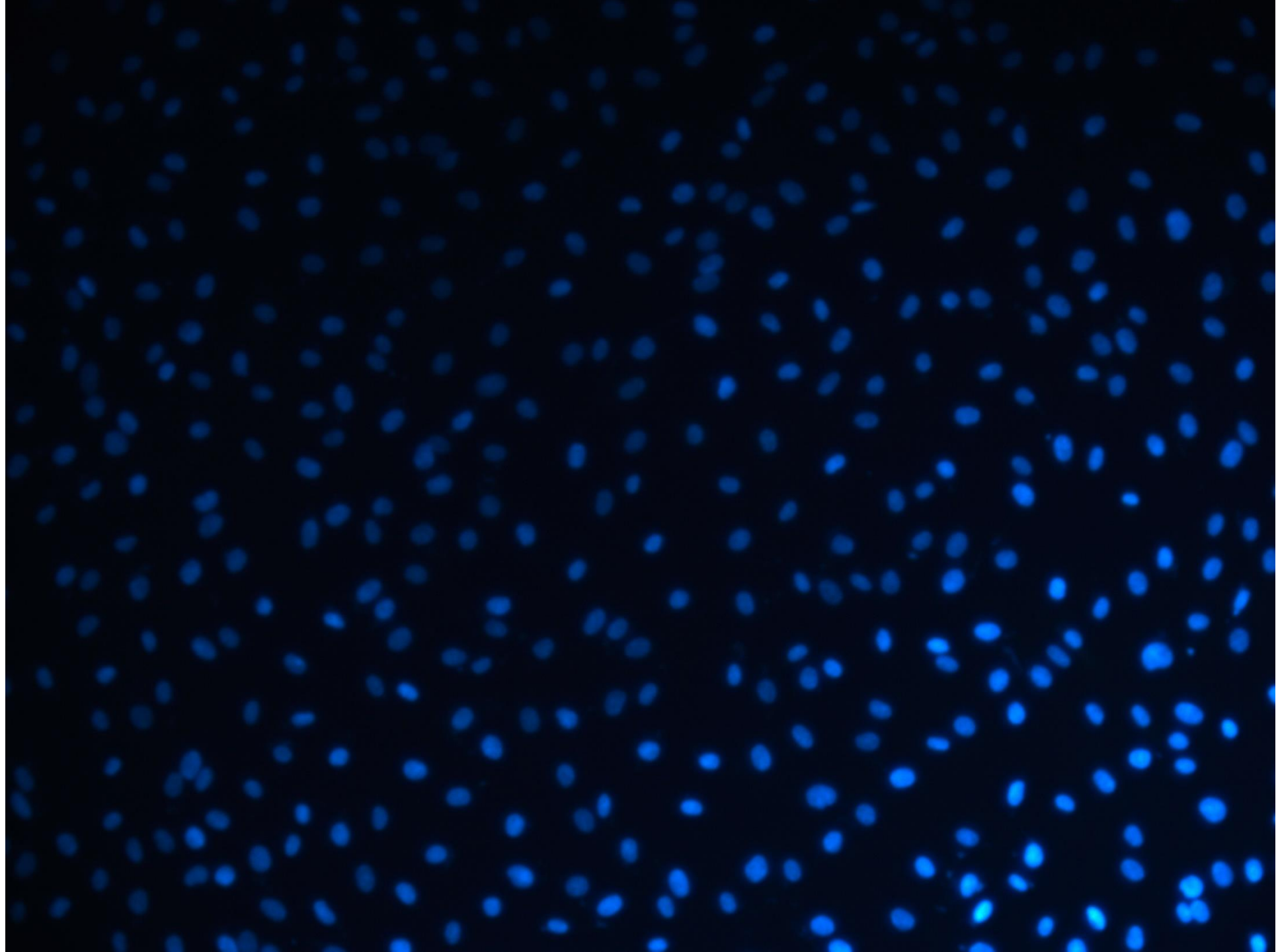

OE-ERCC6L

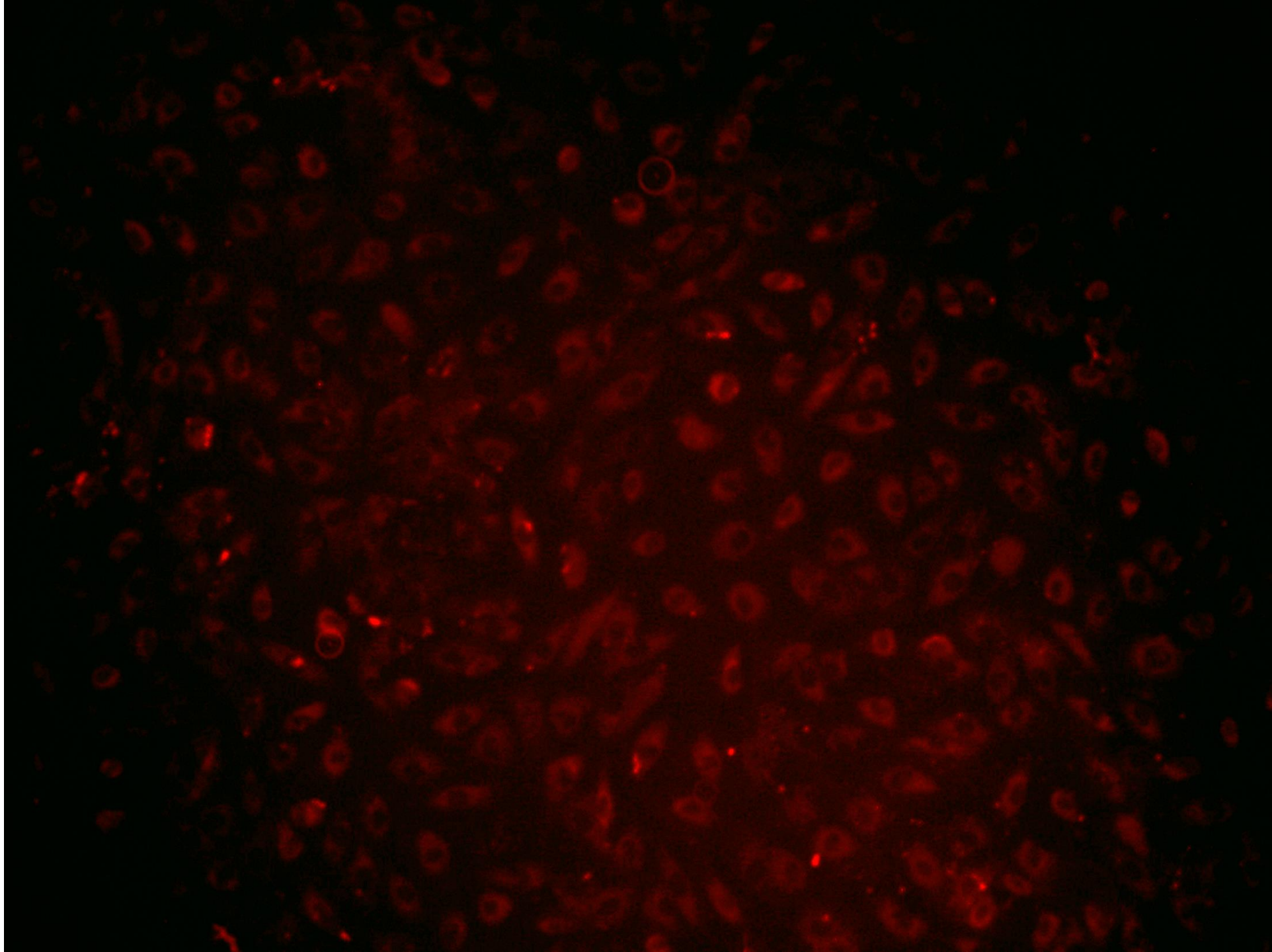

Supplement: Supplementary file 3 — Original Data File [file 41420_2023_1314_MOESM3_ESM.pdf]
